# Effects of Antioxidant Intake on Fetal Development and Maternal/Neonatal Health during Pregnancy

**DOI:** 10.3390/antiox11040648

**Published:** 2022-03-28

**Authors:** Giorgia Sebastiani, Elisabet Navarro-Tapia, Laura Almeida-Toledano, Mariona Serra-Delgado, Anna Lucia Paltrinieri, Óscar García-Algar, Vicente Andreu-Fernández

**Affiliations:** 1 Department of Neonatology, Hospital Clínic-Maternitat, ICGON, BCNatal, 08028 Barcelona, Spain; gsebasti@clinic.cat (G.S.); alpaltrinieri@gmail.com (A.L.P.); 2 Grup de Recerca Infancia i Entorn (GRIE), Institut d’Investigacions Biomèdiques August Pi i Sunyer (IDIBAPS), 08036 Barcelona, Spain; elisabet.navarro@campusviu.es; 3 Faculty of Health Sciences, Valencian International University (VIU), 46002 Valencia, Spain; 4 Institut de Recerca Sant Joan de Déu, 08950 Esplugues de Llobregat, Spain; lalmeida@sjdhospitalbarcelona.org (L.A.-T.); mserrad@sjdhospitalbarcelona.org (M.S.-D.); 5 BCNatal, Fetal Medicine Research Center (Hospital Clínic and Hospital Sant Joan de Déu), University of Barcelona, 08950 Barcelona, Spain

**Keywords:** antioxidant, pregnancy, preterm birth, pre-eclampsia, fetal growth restriction, breastfeeding

## Abstract

During pregnancy, cycles of hypoxia and oxidative stress play a key role in the proper development of the fetus. Hypoxia during the first weeks is crucial for placental development, while the increase in oxygen due to the influx of maternal blood stimulates endothelial growth and angiogenesis. However, an imbalance in the number of oxidative molecules due to endogenous or exogenous factors can overwhelm defense systems and lead to excessive production of reactive oxygen species (ROS). Many pregnancy complications, generated by systemic inflammation and placental vasoconstriction, such as preeclampsia (PE), fetal growth restriction (FGR) and preterm birth (PTB), are related to this increase of ROS. Antioxidants may be a promising tool in this population. However, clinical evidence on their use, especially those of natural origin, is scarce and controversial. Following PRISMA methodology, the current review addresses the use of natural antioxidants, such as epigallocatechin gallate (EGCG), melatonin and resveratrol (RESV), as well as other classical antioxidants (vitamin C and E) during the prenatal period as treatment of the above-mentioned complications. We review the effect of antioxidant supplementation on breast milk in lactating mothers.

## 1. Introduction

During the prenatal period, several complications affecting the mother and the fetus can occur, with consequences for their wellbeing. Preeclampsia (PE), a multisystem progressive disease caused by placental and maternal endothelial dysfunction, usually happens late in pregnancy and complicates 2–8% of pregnancies globally [1]. Moreover, severe and early-onset PE are associated with significant fetal growth restriction (FGR) [2,3], that refers to the fetus that does not grow to its expected biological potential in utero. FGR affects between 3–9% of pregnancies in developed countries and up to 25% in low-middle income countries [4]. According to fetal programming theory, adverse events occurring during critical points of fetal development may cause effects on the offspring and predispose them to chronic diseases later in life [5]. Therefore, placental insufficiency can lead to long-term neurodevelopmental and metabolic diseases, as well as to significant perinatal mortality, especially among prematurely born neonates [6]. However, not only can the FGR predispose the newborn to chronic diseases throughout its life, but prematurity also affects the normal development and the future health of the newborn [5]. Preterm birth (PTB), defined as any birth before 37 completed weeks of gestation, is estimated to be about 11% worldwide [7], i.e., approximately 15 million children are born preterm every year [8]. Prematurity is related to inflammation and oxidative stress pathways [9], and is associated with increased mortality. Many survivors face a wide range of lifetime disabilities, which have financial and organizational consequences on already stretched health services. The World Health Organization (WHO) estimates that more than three-quarters of premature babies can be saved with feasible, cost-effective care [10]. During pregnancy, there is an increase in oxidative stress resulting from placental development, which, under normal conditions, is attenuated by the physiological antioxidant response. The imbalance between oxidative stress and antioxidant response contributes to poor pregnancy outcomes, increasing the risk of PE, FGR and PTB [11,12,13]. Placental ischemia/hypoxia may contribute to oxidative DNA damage due to the release of reactive ROS into the maternal circulation and can act as an enhancer of FGR. In PE, the redox function is also enhanced compared to normal pregnancies [14]. Unbalanced oxidative stress and ROS-produced collagen damage to amniotic membranes are involved not only in PTB but also in premature preterm rupture of membranes (PPROM) [15]. The fetus has to deal with oxidative stress not only in utero, but also at birth, when the newborn has to face a change from an hypoxic environment to a hyperoxic one, which can lead to cytotoxic damage through the creation of ROS and free radicals [16]. Human breast milk contains agents with antioxidant properties, whose concentrations vary during the different stages of lactation, being higher in colostrum. This could impact the antioxidant status of breast-fed infants [17]. Therefore, the intake of antioxidants in breastfeeding mothers could influence the antioxidant concentrations in breast milk, and therefore in breastfed newborns. However, studies on the subject are scarce and the choice of the best antioxidant and best dose to improve neonatal oxidative stress damage remains unknown to date.

Globally, there is an ongoing scientific effort to find solutions to prevent and ameliorate health complications that both mothers and newborns may suffer during the perinatal period. Antioxidant supplementation during pregnancy and breastfeeding could be a promising and cost-effective tool for preventing or treating all these adverse outcomes. However, although the use of antioxidants is widespread and the presence of new emerging antioxidants (such as curcumin, resveratrol or green tea extracts) in the market is increasing, there are few clinical studies in this specific population. Therefore, their effects and safety must be deeply analyzed. The purpose of this systematic review is to analyze the effects of classical and emergent antioxidants in the perinatal period according to the literature published in the last 11 years. Although our review is focused on the use and, more importantly, the safety of these antioxidants in human clinical studies, we also highlighted those studies in different animal models that could complement the information obtained in the clinical area.

## 2. Materials and Methods

**Figure 1:**
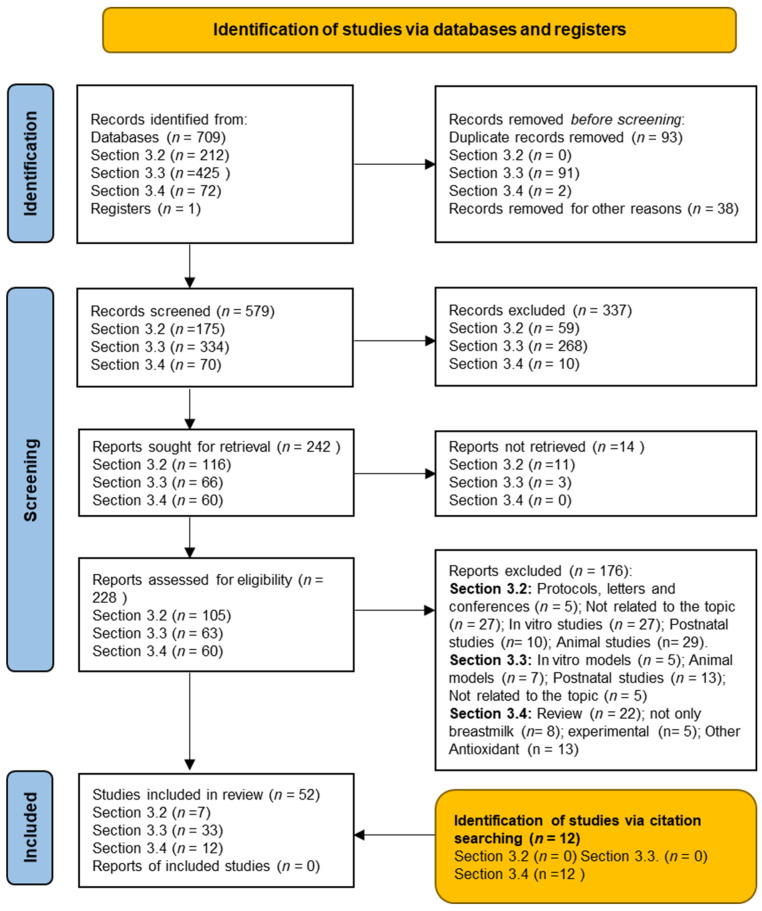
Methodological flowchart based on the PRISMA 2020 update following preferred reporting items for systematic review [20].

The preferred reporting items for systematic reviews and meta-analysis (PRISMA) statement was the methodology selected for the design of the present systematic review [18].The provisional number is 313790. According to the guidelines of the 2009 [19] as well as the update of the 2020 PRISMA statement [20], the research team designed and evaluated the following items: definition of the research question and objectives; bibliographic search; data collection, evaluation, synthesis and comparison; critical evaluation of the scientific papers selected; and finally, analysis of the main findings and conclusions showing the strengths and weakness of the studies evaluated (Figure 1). The objective of the present study was to analyze the therapeutic effects of natural antioxidants in the perinatal period for the treatment of PE, FGR and PTB, as well as their potential benefits in neonatal outcomes through lactation. We did not consider performing a meta-analysis due to the differences in the experimental design depending on the fetal or neonatal stage analyzed, the clinical particularities observed among the studies, and the differences of the molecular mechanism targeted by the diverse antioxidants evaluated in this review, which would generate an important bias in the statistical results.

PubMed (MeSH), Cochrane Central Register of Controlled Trials and Scopus were the electronic databases consulted to collect the data. We performed an initial search using the terms “((antioxidant OR natural antioxidant OR dietary supplement) AND (maternal OR pregnancy OR fetus OR neonate OR newborn OR infant))” to focus the scope of this review on those antioxidants whose effects on perinatal diseases have been analyzed in at least several studies in both human and animal models, since the information available on the therapeutic effects of many antioxidants is still scarce. Once selected, the following descriptors (as MeSH terms or not) were used with the Boolean operators (AND/OR) in multiple combinations (see Supplementary Materials: Methodology): Section 3.2. “((Fetal growth restriction OR intrauterine growth restriction) AND (melatonin OR EGCG OR curcumin OR safranal OR quercetin OR resveratrol)”; ((preeclampsia) AND melatonin OR EGCG OR curcumin OR safranal OR quercetin OR resveratrol))”. Section 3.3. “((obstetric labor OR premature OR preterm delivery OR premature rupture of membrane OR chorioamnionitis) AND (green tea OR EGCG))”; “((obstetric labor OR premature OR preterm delivery OR premature rupture of membrane OR chorioamnionitis) AND (melatonin))”; “((obstetric labor OR premature OR preterm delivery OR premature rupture of membrane OR chorioamnionitis) AND (Zinc))”; “((obstetric labor OR premature OR preterm delivery OR premature rupture of membrane OR chorioamnionitis) AND (vitamin C OR ascorbic acid))”; “((obstetric labor OR premature OR preterm delivery OR premature rupture of membrane OR chorioamnionitis) AND (vitamin E OR alpha tocopherol))”. Section 3.4. “((vitamin C OR ascorbic acid) AND (breast milk OR human milk) AND (neonate OR newborn))”; “((vitamin E OR alpha tocopherol) AND (breast milk OR human milk) AND (neonate OR newborn))”; “((Selenium) AND (breast milk OR human milk) AND (neonate OR newborn))”; “((Zinc) AND (breast milk OR human milk) AND (neonate OR newborn))”; “((melatonin) AND (breast milk OR human milk) AND (neonate OR newborn))”.

Inclusion criteria were papers written in English and Spanish (with no geographical restrictions) published from 1 January 2010 to 1 January 2022; the presence of the selected terms in the title or as keywords; and original research performed in humans. Studies based on animal models were also selected when published studies focused on the therapeutic use of a particular antioxidant to treat a specific perinatal pathology were scarce in humans. The type of experimental designs selected were classical articles, clinical studies, clinical trials, comparative studies, case–control, longitudinal cohort, and cross-sectional and case report studies with a sample size minimum of 10 participants. Exclusion criteria were non-systematic reviews, lack of a control group with no antioxidant treatment, interventions using drugs or other antioxidants that could interfere with the effects of a specific antioxidant on these pathologies, the presence of genetic or other diseases in the mother or neonate that could interfere with the alterations analyzed in this review, and papers whose output variables were not related to treatments with antioxidants during the perinatal period.

The researchers E.N.-T. and V.A.-F. for Section 3.2, G.S. and V.A.-F. for Section 3.3, and L.A.T. and M.S.-D. for Section 3.4 performed the initial selection of original manuscripts by screening titles and abstracts and creating a reference list of papers for the topics evaluated in the present review. Three investigators (E.N.-T., G.S. and V.A.-F.) conducted each stage of the study selection, deleted duplicate inputs and reviewed studies as excluded or requiring further assessment. All data were extracted by two investigators (G.S. and E.N.-T.) and cross-checked by the other two investigators (E.N.-T. and V.A.F.). In the case of discrepancies in the selected studies, we opted for reconciliation through team discussion. Moreover, the reference list of selected papers was checked manually to include some high-impact additional studies from the bibliography of the original papers or meta-analyses that were relevant to the topics addressed (Figure 1). The information evaluated from each study was: first author, year of publication, experimental design (treatment duration and study groups), number of participants in the treated and control group; main outcomes/findings; conclusions; strengths and limitations (including biases). The eligibility criteria followed the PICOS approach (patient, intervention, comparators, outcome and study design). Population: fetuses or newborns diagnosed with some of the perinatal diseases included in this study (preeclampsia, fetal growth restriction, prematurity or other neonatal outcomes); intervention: any dose of antioxidant selected for this review; comparators: if applicable placebo; outcome: the primary outcome was the response of the patients or animals with some developmental alteration to the antioxidant treatment; changes in levels or expression of molecular biomarkers related to diverse physiological functions during development. All authors performed a critical appraisal of the studies selected following the inclusion criteria, also analyzing the methodology and key results.

The outputs evaluated following PRISMA were heterogeneous by the need to include animal models as well as human studies; the different populations analyzed (fetuses, newborns or infants); the small sample size observed in many of these studies; and the few randomized trials using some antioxidants as pharmacological treatment of these perinatal alterations. Finally, the studies identified through databases searching, selection after meeting the inclusion criteria and the application of the exclusion criteria were: Section 3.2 (7); Section 3.3 (33); Section 3.4 (12) (Figure 1).

The quality of evidence was based on the GRADE approach (Grades of Recommendation, Assessment, Development and Evaluation), which describes four levels of quality: high, moderate, low and very low [21,22]. The quality of evidence was judged (Supplementary Materials Table S1) by all the authors (at least 2 authors independently for each section), focusing on the experimental design of the studies, number of subjects, risk of bias, inconsistency, indirectness, imprecision and relative or absolute effects observed. Disagreements were resolved through a consensus-based discussion.

## 3. Results

### 3.1. Characteristics of the Studies Included

After a bibliographic search, 709 published articles were identified in the databases indicated in the Material and Methods section. Once duplicate papers were deleted, the abstract and title of the potentially relevant 579 articles were reviewed, and 337 references were eliminated. Of the 579 screened studies, 242 were sought for retrieval. The full text of the remaining 228 references, once 14 references were not retrieved, was carefully analyzed and reviewed. A total of 176 articles were removed following the exclusion criteria and because they did not meet inclusion criteria due to lack of a controlled design, not meeting the setting characteristics or not meeting the review objective. Finally, 52 studies were eligible and included in this systematic review, as shown in the flowchart of Figure 1. An additional 12 studies were included via citation searching.

### 3.2. Antioxidants’ Use during Pregnancy: Effects on Preeclampsia and Fetal Growth Restriction

To date, 7 studies have been carried out with natural antioxidants: curcumin, EGCG, melatonin and resveratrol (RESV). Their use is gaining popularity. Most of these studies were conducted in China, Australia and Brazil, and only one of them (a pilot study) was conducted in women with severe early onset FGR. Although we also included safranal in the search, due to its great antioxidant potential [23], we did not find any clinical studies with this antioxidant in this population. A detailed summary of the main results, the population studied, and the treatments administered on PE and FGR are shown in Table 1.

#### 3.2.1. Curcumin

Curcumin is a polyphenolic substance generally recognized as safe (GRAS), which comes from the rhizomes of *Curcuma longa* (turmeric), and has been shown to have antioxidant and anti-inflammatory properties in humans [24]. In addition, it is emerging as a promising adjuvant in the fight against COVID-19 due to its potential to activate NFE2-related factor-2 (Nrf2) and decrease inflammatory cytokines [25]. The therapeutic effect of curcumin to counteract certain complications during pregnancy, such as PE and FGR, has been tested in vitro studies, showing an increase in angiogenesis and a decrease in oxidative stress through activation of the Nrf2 signaling pathway [26,27]. To date, we have only found one human study using curcumin in women with PE; however, the variables determined and treatment period were limited [28]. The authors measured levels of cyclooxygenase-2 (COX-2), associated with increased vasoconstrictor thromboxane [29], and IL-10 in the blood of 47 women before and after undergoing cesarean section and after taking 100 mg curcumin or a placebo (Table 1). The authors found no significant differences in these variables after taking the polyphenol. Regarding FGR, we have not found human studies in which curcumin was used for its prevention. However, the beneficial effects of curcumin on PE and FGR have been observed in animal studies. Gong et al. [30] observed significant improvement of hypertension and proteinuria in a rat model of PE, obtained by intravenous administration of LPS (0.5 mg/kg LPS (*Escherichia coli* serotype 0111:B4) on gestational day (GD) 5. The LPS-curcumin-treated group reached control rat levels. In addition, levels of Toll Like Receptor 4 (TLR4) and inflammatory factors, such as Nuclear Factor κB (NF-κB), IL-6 and Monocyte Chemoattractant Protein-1 (MCP-1), were also significantly decreased compared to the untreated PE group. The authors also observed that the weight of the offspring in the PE group was significantly lower than in the control group. However, the weight of the offspring in the curcumin-treated PE group did not differ from the control group, demonstrating a protective effect on this variable. For the same year, Zhou et al. [31] performed a similar experiment in mice using intravenous administration of LPS: 10 μg/kg and 40 μg/kg LPS (*Escherichia coli* serotype 0111:B4) from GD 13.5 until GD 16.5. The authors tested a curcumin concentration 3 times lower than the one used by Gong et al. [30], and the results revealed a significant improvement of hypertension and proteinuria in the curcumin-treated PE group, reaching control group levels in the latter case. In addition, curcumin also significantly decreased Lipopolysaccharides (LPS)-generated inflammation through upregulation of phosphorylated Protein kinase B (Akt) and significantly increased the weight and number of fetuses to control levels. No developmental alterations in fetuses were observed in any of the previous studies.

The administration of different doses of this hydrophobic polyphenol by gavage has also been tested in mouse models of fetal restriction by low-protein (LP) diets. In the study carried out by Qi et al. [32], it was observed that a dose of 400 mg/kg/day generated significantly greater fetal gain compared to doses of 100 mg/kg/day and both doses significantly increased fetal and placental weights compared to the untreated LP group. Regardless of dose, in the placenta curcumin reduced levels of oxidative stress marker malondialdehyde (MDA) and of apoptosis to control group levels (without FGR). In contrast, these levels were significantly higher in the untreated LP group. Remarkably, the highest curcumin concentration also restored the percentage of blood sinusoid area in the placenta to control group levels. However, the bioavailability of the antioxidants was not measured in these animals.

#### 3.2.2. EGCG

Epigallocatechin-3-gallate is the most abundant catechin in green tea and has been extensively studied in numerous clinical studies for the treatment of diabetes, appetite control, weight loss or cognitive improvement [39,40,41,42]. Moreover, its bioavailability has been tested with different nutritional strategies, facilitating the most appropriate choice based on the purpose of the study [43]. However, there are very few clinical studies on the effect of EGCG on gestational complications, such as diabetes mellitus [44]. To date, only one study with EGCG in the PE has been identified [33]. This work (Table 1) shows that the use of EGCG in women with severe PE together with nifedipine, one the first-line drugs to treat high blood pressure, leads to a significant decrease in blood pressure until normal values compared with nifedipine alone (with the mean difference of 14.1 min). This antioxidant also increased the interval before a new hypertensive crisis [33]. Although there is insufficient evidence of the effect of EGCG on PE in humans or in animal models (with no studies in the latter), it has recently been shown that EGCG exerts a protective role against endothelial dysfunction and enhances the anti-angiogenic status in hypoxic trophoblast cells. The protective effect of EGCG appears to be due, in part, to the inhibition of the expression of the high mobility group box 1 (HMGB1), a late inflammatory factor released by trophoblasts during hypoxia that induces endothelial damage [45]. In addition, the authors observed that this antioxidant significantly increased cell viability under hypoxic conditions. These results were dose-dependent, demonstrating the usefulness of EGCG against the main causes of PE, trophoblast apoptosis and endothelial dysfunction [46,47].

Regarding FGR, although it has been shown in mice that antenatal ECGC can counteract the fetal restriction generated by other reasons, such as alcohol consumption [48], its effect in humans has not been tested. The postnatal consumption of this antioxidant in mice subjected to FGR decreases fatty acid synthesis through the Ampk/Srebf1 signaling pathway and significantly reduces cholesterol and triglyceride levels in the liver compared to the untreated group [49]. The relationship between low birth weight and hypertriglyceridemia has been demonstrated [50,51], so postnatal use of EGCG could help to reduce cardiovascular risk in this population. However, further studies must be conducted.

#### 3.2.3. Resveratrol

RESV is a polyphenol extracted from fruit, such as grapes and cranberries, and has been reported to be safe for human consumption at doses up to 5 g per day [52]. In addition, it is able to cross the placenta in both rats and pregnant nonhuman primates [53,54].

As with EGCG, RESV has been used together with nifedipine in women with severe PE [35]. The results (Table 1) showed a significantly faster blood pressure decrease than with nifedipine alone, similar to those obtained with EGCG [33]. RESV, such as EGCG, also significantly increased the time interval before a new hypertensive crisis and none of them produced adverse effects at the neonatal or maternal level. However, the RESV concentration used was lower (50 mg/capsule) compared to EGCG (100 mg/capsule). Co-treatment with RESV in endothelial cells (HUVECs) treated with serum from PE pregnant women restored the levels of heme oxygenase-1 (HO-1) and nitric oxide (NO) markers [36], key factors for placental vasculature and endothelial protection [55,56]. Thus, the author published a pilot clinical study in PE patients years later. They showed that patients’ serum after grape juice intake significantly decreased HO-1 and glutathione (GSH) levels in HUVECs, compared to juice pre-ingestion levels [37], acting on other mechanisms compared with cotreatment of plasma from PE and RESV. Similar to the previous study with pure RESV, the treatment increased NO levels and did not alter ROS levels in these cells. Remarkably, the same concentration of serum from grape juice intake decreased the expression of the Antioxidant Response Element (ARE) in HUVECs by about 69%, in contrast to pure RESV intake, which boosted its activity by 78%. All these results show how the different active components of grape juice can act differently from RESV to exert a beneficial effect in the treatment of PE [37].

Despite few human studies with RESV in PE, its effect has been extensively studied in murine models. Dietary supplementation with RESV in a genetic model that mimics the phenotypic characteristics of PE and FGR has shown a significant increase in uterine artery blood flow velocity and fetal weight [57]. A significant decrease in blood pressure, oxidative stress and apoptosis has also been seen in trophoblasts derived from placentas of PE rats treated with RESV [58], as well as a significant improvement in placental epithelial characteristics [59]. However, unlike the previous study, fetus birth weight did not change compared to the non-treated group. Subcutaneous supplementation with RESV through subepidermal patches has also been tested in ewe. Results showed a significant increase in uterine artery blood flow and fetal weight, although maternal RESV treatment had no effect on placental weight [60]. All of these data present RESV as a promising therapeutic strategy for PE and FGR, although clinical studies in PE and FGR with RESV are currently very limited.

#### 3.2.4. Melatonin

Melatonin, synthesized mainly in the pineal gland, can easily cross the placenta and exert its antioxidant action, regulate cell proliferation in the fetus, and maintain pregnancy [61,62]. A recent meta-analysis showed that its concentration is significantly lower in women with PE and its levels correlate with the severity of the disease, being significantly lower in severe PE than mild PE [63]. Likewise, in the case of placental insufficiency, melatonin 1A and 1B receptors are significantly less expressed in the placental tissue of mothers of FGR fetuses [64]. In addition, melatonin and placental growth factor (PLGF) in the umbilical blood were significantly lower in this group compared to normal pregnancies [65,66]. Our search only obtained two studies that focused on PE and the use of melatonin (Table 1). The first is a protocol for a phase I pilot clinical trial (the PAMPR Trial) in women with early-onset pre-eclampsia [67], and their results were published five years later [34]. The use of extended-release tablets in 20 women with PE prolonged the interval from diagnosis to delivery by almost one week, although no difference in average mean arterial blood pressure was observed. Moreover, the melatonin group required less antihypertensive medication compared to historical controls [34]. Despite the limited clinical data linking melatonin to PE, extensive animal studies have demonstrated that the use of melatonin as an adjuvant in high-risk pregnancies is very promising. It has been shown recently that melatonin exerted neuroprotective effects and increased PLFG levels, a key molecule in embryonic angiogenesis and vasculogenesis, and reduced placental tumor necrosis factor-alpha (TNF-α) levels, exerting anti-inflammatory effects in a rat model of PE [68]. Melatonin also increased the transforming growth factor-beta (TGF-β) levels in the fetal brain, promoting the maturation of newborn neurons and improving brain weight. Melatonin also decreased hypertension, placental IL-6 expression, oxidative stress and proteinuria in murine models of PE [69,70]. In offspring, maternal melatonin treatment ameliorated fetal heart damage caused by reduced uterine perfusion pressure (RUPP) [71]. Melatonin also had a global epigenetic effect during nephrogenesis and restored the ADMA-NO balance in the kidney in a rat model [72,73].

FGR is known to be associated with structural deficits of the brain, such as fragmentation and disorganization of the cerebral white matter tracts and decreased myelination [38]. Taking into account the relationship between FGR and oxidative stress, melatonin could help in the correct development of brain structure and function in the fetus. To date, only one pilot study relating to the neuroprotective effect of melatonin in FGR has been published [38]. The authors observed almost half the concentration of MDA in placentas from mothers who had taken 8 mg of melatonin during the last weeks of their pregnancy. In addition, melatonin was well tolerated, and no adverse effects were observed (Table 1). Although the sample size of this study was small (12 patients), several animal studies support the use of this antioxidant for fetal restriction [74,75,76,77,78], and its effect in preventing oxidative stress-related FGR is greater than other compounds, such as sertraline or diazepam [79]. The intravenous administration of maternal melatonin in lambs significantly improved neonatal behaviors, lipid peroxidation, organization and density of certain brain areas and also protected the blood–brain barrier [38,80]. No prevention of pulmonary alveolar disruption was observed [81]. Despite these facts, there are indications that melatonin may cause decreased neonatal biometric parameters in pregnancies of sheep exposed to chronic hypoxia due to high altitude, so further studies are needed in this specific population [82].

Currently, the “PROTECT-ME” study (ACTRN12617001515381), a triple-blind, randomized, parallel group, placebo-controlled trial, is trying to determine whether antenatal maternal melatonin supplementation improves neurodevelopmental outcomes at 2 years of age in children affected by FGR. In this trial, mothers received 30 mg/day of melatonin antenatally compared to the placebo group. No results have yet been published yet [83].

**Figure 2:**
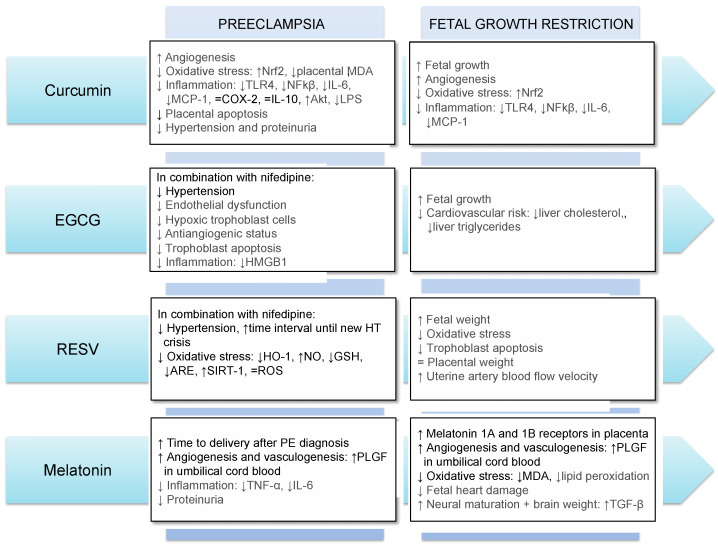
Main effects of the use of curcumin, EGCG, resveratrol and melatonin in preeclampsia and fetal growth restriction. Results in gray and black refer to those obtained in animal models and human, respectively. Abbreviations: Akt: Protein kinase B; ARE: antioxidant response element; COX-2: Cyclooxygenase-2; GSH: glutathione; HMGB1: high-mobility group box 1; HO-1: heme oxygenase-1; HT: hypertension; IL-6: interleukin-6; IL-10: interleukin-10; LPS: lipopolysaccharides; MCP-1: monocyte chemoattractant protein-1; MDA: malondialdehyde; NFkβ: nuclear factor κB; NO: nitric oxide; Nrf2: NFE2-related factor-2; RESV: resveratrol; ROS: reactive oxygen species; SIRT-1: sirtuin-1; TGF-β: transforming growth factor-beta; TLR4: Toll Like Receptor 4; TNF-α: tumor necrosis factor-alpha; PLGF: placental growth factor; ↓: decrease; ↑: increase; =: no difference.

The main results obtained so far regarding the use of antioxidants in FGR and PE in humans and animals are summarized in Figure 2.

### 3.3. Effects of Antioxidants on Prematurity

The global community is concerned about the burden associated with the high number of PTB and prematurity-related complications [84]. Currently, specific treatments are not always successful in delaying birth up until term age, so new strategies for preventing PTB may be useful to avoid morbidity and mortality associated with prematurity. As mentioned above, unbalanced oxidative stress during gestation may cause PPROM [15] and PTB [9]. Therefore, supplementation with classic antioxidant agents (vitamin C or vitamin E), or some trace elements with antioxidant properties, such as zinc, may be considered an option in the prevention of prematurity. In addition, other novel antioxidant strategies, such as tea or melatonin, are being considered. In this review, we selected 33 studies that analyzed the effect of the above-mentioned antioxidants on prematurity. Table 2 summarizes the main results according to the objective of the study and the antioxidants evaluated.

#### 3.3.1. Effects of Vitamin C and Vitamin E on Prematurity

The effects of vitamin C on prematurity were evaluated in 10 studies. Consumption of vitamin C-rich products did not decrease the risk of PTB [97] and, in some cases, it was associated with a higher risk [85]. Nevertheless, vitamin C deficiency during pregnancy was associated with increased risk of PPROM (*p* < 0.05) [15,86,87,88,94,95], as well as a shorter latency period before birth in women with PPROM (*p* < 0.001) [92]. Only one study showed a higher risk of PPROM associated with higher dietary vitamin C intake in the first and second trimester of pregnancy [93]. ROS could produce collagen injury to chorioamniotic membranes, leading to PROM. In addition to its antioxidant activity, vitamin C is involved in collagen metabolism and plays an important role in the integrity of amniotic membranes. Therefore, vitamin C supplementation may be a promising therapy for the maintenance of the integrity of amniotic membranes and the prevention of PPROM. A daily dose of 100 mg vitamin C during pregnancy, as can be seen in the randomized controlled trial (RCT) of Ghomian et al. [86], could be a good option to prevent PPROM. More studies analyzing plasmatic levels of vitamin C according to different doses are needed to assess the relationship between vitamin C and PTB or PPROM.

Vitamin E antioxidant power relies on its role as a chain-breaking antioxidant and its lipid peroxyl scavenger function [9]. Nine studies assessed the relationship between vitamin E intake and prematurity. With respect to PPROM, dietary vitamin E was not associated with a reduction in PPROM [15,93]. However, the RCT presented by Gungorduk et al. [92], showed a beneficial effect of vitamin E supplementation with 400 IU in combination with 1000 mg vitamin C in the reduction of the latency period to birth in PPROM patients. When analyzing PTB, the results were discordant. 3 studies [90,96,97] showed a preventive effect of vitamin E on PTB. The supplementation with a daily dose of 450 mg vitamin E in the Hungarian population was associated with a 30% reduction in PTB [90]. In addition, Koenig et al. [96] showed an inhibition of premature cervical remodeling in women with a high intake of vitamin E, which could explain the reduction in PTB. Conversely, other studies [89,91,94,95] did not show an association between vitamin E and PTB prevention. According to the studies reviewed, vitamin E alone seems to have no effect on PPROM prevention, and it remains unclear whether vitamin E supplementation during pregnancy may be beneficial in PTB prevention. Well-designed studies are necessary to evaluate the role of vitamin E in PTB.

#### 3.3.2. Zinc Supplementation on Prematurity

A number of micronutrients, including trace elements, such as zinc, are known as antioxidants or essential cofactors for antioxidant enzymes [103]. Zinc is involved in DNA synthesis as a component of nucleic acids and several enzymes [104]. Adequate zinc intake is essential for normal pregnancy development [100]. Zinc deficiency during pregnancy has been linked to PTB and other adverse obstetric outcomes; therefore, zinc supplementation during pregnancy is considered in some populations [100]. Seven studies analyzed the effect of zinc supplementation on prematurity. The RCT completed by Nossier et al. [100] showed a statistically significant reduction in PTB in mothers supplemented daily with 30 mg of zinc (1%) compared to controls (10%). However, other authors reported opposite results. According to Zahiri et al. [101], the supplementation with 15 mg of zinc daily did not reduce the risk of PTB. Optimal zinc levels also did not reduce PTB in Japanese women who gave birth prematurely without PPROM [103], although in this case a beneficial effect of zinc was found in reducing PPROM (*p* < 0.01). In addition, Nga et al. [99] also found similar rates of PTB in low-income populations supplemented with zinc compared to controls, as well as in women with dietary interventions and in vitro fertilization [98], or previous bariatric surgery [102]. Oxidative stress-induced DNA damage could be reversed by zinc supplementation [103], but current studies do not support the use of zinc in PTB prevention. Further studies with supplements of at least 30 mg of zinc should be conducted to clarify zinc’s role in reducing PTB and PPROM.

#### 3.3.3. Black and Green Tea

Tea is widely consumed worldwide. Tea catechins have beneficial effects on health due to their antioxidant properties [106]. However, during pregnancy, caffeine content has been associated with PTB [107]. Seven studies evaluated the effect of tea consumption on PTB. In 4 studies [105,106,108,110], tea intake during pregnancy (green or black tea) was associated with increased risk of PTB (*p* < 0.05). In the remaining 3 studies [107,109,111], there was no statistically significant association between tea drinking and PTB, but lower tea intake was reported in these studies. When evaluating the effect of tea on PTB, all studies analyzed consumption through the ingestion of cups of tea or food frequency questionnaires (FFQ) without differentiating the tea components, so the results are confusing. Probably, the association between tea intake and PTB found in some studies is due to caffeine content. Clinical trials are needed to evaluate the real effect of catechins in tea on prematurity prevention.

#### 3.3.4. Use of Melatonin on Prematurity

Melatonin has antioxidant effects, such as scavenging free radicals and enhancing antioxidant mechanisms, as well as anti-inflammatory effects, which can be beneficial in preventing PTB [113]. Studies in murine models [113,114] showed the effect of melatonin on antioxidant systems, producing a statistically significant reduction in NOS and iNOS activity [113], as well as an increase in Nrf2 and SIRT-1 levels [114]. These promising results make translation to humans possible. Results reported in two human studies [112,115] showed an association between low melatonin levels and increased risk of PTB (*p* < 0.05). However, Specht et al. [116] did not find an association between night-shift working and prematurity. More human studies are needed to elucidate the effects of melatonin on antioxidant systems for the prevention of prematurity.

**Figure 3:**
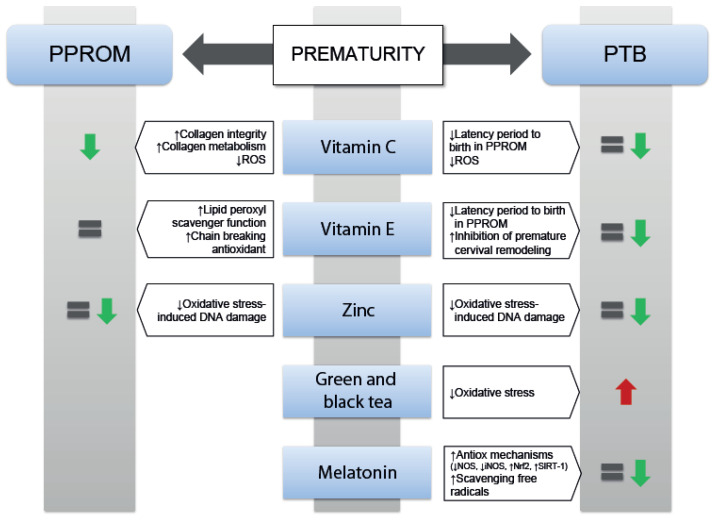
Main effects of the use of vitamin C, vitamin E, zinc, tea and melatonin in prematurity. Green arrow means reduction PPROM/PTB rates, red arrow means increase in PTB, and equal sign means no changes in PPROM/PTB rates. Abbreviations: iNOS: inducible nitric oxide synthase; NOS: nitric oxide synthase; Nrf2: NFE2-related factor-2; PPROM: preterm premature rupture of membranes; ROS: reactive oxygen species; SIRT-1: sirtuin-1; ↓: decrease; ↑: increase.

The main results obtained so far regarding the use of antioxidants in prematurity in humans and animals are summarized in Figure 3.

### 3.4. Effects of Antioxidants in Human Milk and Neonatal Outcomes

Clinical trials about antioxidant supplements in lactating mothers are scarce in the literature. Most published studies are about the concentrations of antioxidants in breast milk. During the first steps of development, the embryo is exposed to low levels of oxygen. Trophoblasts are more susceptible to hyperoxia and variable amounts of oxygen. Low oxygen levels enhance the proliferation of trophoblasts, promoting maintenance of cell self-renewal and regeneration [117]. The oxygen concentration vary during the different phases of gestation, from up to 20 mmHg in the first trimester to ~60 mmHg in the second trimester and it progressively declines to ~40 mmHg at term of gestation, so the placenta is highly sensitive to changes in oxygen levels and oxidative stress [118]. After delivery, environmental oxygen becomes predominant, being potentially toxic for infants because of the creation of ROS and free radicals [16]. Human milk contains many compounds with antioxidant properties due to their chemical structure, which promptly neutralizes free radical groups and oxidative stress, modulating redox signaling. Breast milk exerts antioxidant function as a protective mechanism against infections and diseases in order to maintain a balance between ROS and antioxidant concentrations for a healthy stable status in infants [119]. The immunomodulatory and anti-inflammatory characteristics of breast milk are performed by polyunsaturated fatty acids, growth factors, nucleotides, cytokines and antioxidants. The main antioxidant components derived from the diet in breast milk are vitamin C, α-tocopherol (vitamin E) and β-carotene (vitamin A) [16].

#### 3.4.1. Vitamin C and Vitamin E in Lactation

Maternal diet enriched in vitamin C influences the concentrations of such antioxidants in breast milk, and high concentrations are associated with low risk of atopy in infancy, a disease characterized by increased oxidative stress [120].

Daily supplements of 500 mg of vitamin C and 100 mg of vitamin E consumed by lactating mothers with breastfed neonates for 30 days considerably improved the antioxidant power of the breast milk. They also enhanced the antioxidant content and scavenging capacity of infant urine, emphasizing the importance of the transport of antioxidants from the breast milk to the infants and the elimination of unnecessary quantities in the infants’ urine [121]. Vitamin C supplements may increase the levels in human milk in women with low content at baseline, suggesting an intrinsic mechanism related to the regulation of ascorbic acid secretion and saturation [122]. Conversely, an experimental study demonstrated that supplements with Vitamin C and iron added to human milk increased DNA damage if compared to these supplements given alone, so iron and vitamin C may be separated especially in preterm babies to avoid radical-induced damage, but more studies are necessary to demonstrate it [123].

Vitamin E, essential for the development of the immune system, lungs, vascular system and mental development, decreases with the evolution of lactation and is scarce in mature milk [124]. The average proposed intake of vitamin E (α-tocopherol) for children between 0–6 months is in 4 mg/day [124], its intake increases the concentration of α-tocopherol in colostrum and the antioxidant capability in the newborn [125]. Supplements with α-tocopherol to mothers of preterm babies increased their levels in the colostrum and transitional milk, but not in the mature milk, so the effect doesn’t seem to be prolonged [126]. A possible explanation may suggest that this antioxidant plays a significant role in lipid metabolism, so its supplementation enhances the synthesis of fatty acids by the mammary gland in the first few days after delivery and is more present in colostrum than in mature milk [127]. Therefore, the antioxidant property prompted by these 2 vitamins is more active during the first days after delivery, the most critical period to counteract oxidative stress in the newborn.

#### 3.4.2. Selenium and Zinc

Selenium and zinc are essential trace elements that protect against oxidative stress and have immunomodulatory properties. Low levels of selenium are linked to increased oxidative lung disease [128]. No significant differences were found in selenium status at birth between appropriate for gestational age (AGA) and small for gestational age (SGA) babies. There was a variability in selenium status related to postnatal age and it was affected by the type of feeding, being breast milk the best source of selenium [129]. Zinc supplements in lactating women increased breast milk zinc levels and maternal body stores but it did not impact the infants’ physical growth. This suggests that zinc stores were adequate, whether their mothers were not supplemented, probably due to the regulation mechanisms of intestinal zinc retention to meet growth demand [130]. In preterm babies, human milk fortifiers containing trace elements, calcium, phosphorus and proteins did not impact serum zinc levels at 3 and 6 weeks, and did not induce more weight gain, but selenium concentrations resulted in higher [131]. These results may be explained by the different factors that influence zinc homeostasis by modulating intestinal absorption and urine excretion, leading to different serum zinc levels. Moreover, zinc concentration in maternal milk is extremely irregular, usually unknown and drops during lactation.

#### 3.4.3. Use of Melatonin in Lactation

In breast milk, melatonin shows an evident circadian rhythm, as seen by elevated levels during the night and untraceable levels during the day. This melatonin pattern in human milk could be necessary to inform the breast-fed infant about the moment of the day; this information could also help the infant to establish a sleep-wake pattern until the development of the mature daily rhythm [132]. Qin et al. demonstrated that melatonin showed a circadian pattern in both preterm and term breast milk among the different lactation phases. Compared with term milk, preterm milk showed a greater top concentration of melatonin in every tested lactation phase; this may be an advantage for preterm infants during the first days of life because of their immature neurological system [133].

Melatonin levels in human colostrum showed daily fluctuation and enhanced phagocytic properties of colostrum cells against bacteria. Therefore, melatonin promotes the cellular oxidative pathways of colostrum phagocytes. Therefore, melatonin enhances colostrum’s property to safeguard neonates against bacterial infections, supporting newborn’s adjustment to environmental variations, leading to the formation of metabolic antioxidant pathways across breastfeeding [134].

The main results obtained so far regarding the use of antioxidants in lactating mothers are summarized in Table 3.

## 4. Conclusions

The present work carried out an in-depth analysis of the effects of different antioxidants on fetal development, maternal health during pregnancy and neonatal health. It analyzed widely studied antioxidants, such as vitamin C and E, and new emerging antioxidants, such as curcumin, resveratrol and EGCG.

Antioxidants could have potential beneficial effects on pregnancy complications such as PE, FGR and PTB. Their principal mechanisms comprise anti-inflammatory, anti-apoptotic and anti-angiogenic effects, as well as the ability to restore redox homeostasis [136]. For these reasons, the interest in their use during the perinatal period has grown.

Antioxidants have shown promising results for PE and FGR. Curcumin exerts an anti-inflammatory effect through inhibition of NF-κβ [137], a COX-2 activator, explaining its possible therapeutic effect against PE, where higher placental levels of thromboxane related to COX-2 expression (a potent vasoconstrictor) are evident [138]. Currently, the only published work is based on a single dose, once and before cesarean section, without satisfactory results [28]. However, further studies are needed to evaluate both the effective dose and the form of administration to enhance its bioavailability and oral and gastrointestinal absorption. Micellar systems, or hydrophilic nanoparticles, could increase curcumin concentration up to 15–20 fold [139]. Other forms of administration, such as nebulized curcumin, have also shown excellent results in improving postnatal pulmonary disorders due to fetal restriction in rat pups [140]. Thus, prenatal studies in animal models show promising results against FGR, protection against placental apoptosis and poor nutrient transportation in placenta due to the loss of blood sinusoid area [32]. However, although its safety has been proven with no adverse effects on reproductive performance or embryos in animal models [141,142], curcumin has to be extensively studied before conducting groundbreaking studies on the use of curcumin on FGR, due to the lack of data on its use in this population.

In PE, EGCG and RESV have also shown promising results, enhancing the efficacy of nifedipine [33,35]. Studies showed similar results in terms of the time needed to return to normal blood pressure values and the number of doses needed. However, despite the fact that these compounds have a long history of safe use, few clinical studies in pregnant women have been published. EGCG and RESV have been shown not only to improve severe PE, but also the metabolic profile in overweight and diabetic pregnant women [44,143].

Although the use of these antioxidants as a single therapy is still far away, these two studies create a precedent for the use of antioxidants as a coadjuvant against pregnancy complications. In this way, antihypertensive efficacy could be increased without resorting to drugs with more side effects, uncomfortable (non-oral) administration, or more expensive. Grape juice, also rich in RESV and other bioactive compounds, is more accessible and cheaper than its pure form and has been shown to balance NO levels [144], whose decrease in serum increases the risk of PE [145]. However, its high sugar content must be taken into account. Melatonin has also achieved encouraging results in animals and its safety has been tested in women with PE [34] and on FGR [38]. Recently, it has been published that Melatonin-MT1 signal is essential for endometrial decidualization and that melatonin could reverse the inflammation and decidualization resistance induced by LPS [146]. However, there is no study or research about their long-term effects, and it could be contraindicated in populations living at high altitudes [82]. However, the use of melatonin, especially in extended-release tablets to ensure sustained high melatonin levels over time, could decrease the need for antihypertensive medication [34], which is directly related to FGR [147].

Oxidative stress is considered one of the pathophysiological factors related to sPTB, a worldwide problem with consequences for newborn health, so antioxidant strategies could be a feasible option for prematurity prevention. Different antioxidants are considered in this review to modulate oxidative stress linked to prematurity. Classical antioxidants, such as vitamin C or vitamin E, have been tested in different studies to evaluate their effect on PPROM and PTB reduction. Vitamin C is involved in collagen metabolism and integrity, so its use prevents ROS-induced collagen injury to chorioamniotic membranes, reducing PPROM, as shown in different studies [15,86,87,88,94,95]. In the case of vitamin E, its lipid peroxyl scavenger function and chain-breaking antioxidant activity [9] could increase the latency period to birth in PPROM cases [92]. Additionally, vitamin E inhibits premature cervical remodeling, which can lead to PTB [96]. However, not all studies evaluating the effect of vitamin C and vitamin E on PTB have shown a reduction in prematurity. Well-designed studies with known safe doses of vitamin C and vitamin E are needed to evaluate the role of these antioxidants in PTB. Regarding zinc, studies with different daily supplementation doses have been carried out with different results. Supplementation with low doses of zinc has shown no effect on prematurity [99,101,103], while doses of at least 30 mg of zinc appear to be effective in reducing PTB rates [100] through the inhibition of oxidative-stress induced DNA damage [103].

New antioxidant strategies, such as green and black tea or melatonin, have also been evaluated for prematurity prevention. In the case of tea, despite its antioxidant power related to catechin content [106], results from different studies showed an increase in PTB rates [105,106,108,110], probably due to caffeine content in tea [107]. It would be interesting to design studies using catechins alone to evaluate the real effect of this antioxidant on prematurity. Nevertheless, melatonin has shown promising results in murine models [113,114]: reduced NOS and iNOS activity and increased Nrf2 and SIRT-1 levels found in these models, in combination with the free radical scavenger action, lead to a reduction in PTB. These promising results have to be translated to the human population in order to evaluate the effects of melatonin on antioxidant systems for prematurity prevention.

Once delivery occurs, the newborn replaces an intrauterine environment (with about 11% air saturation) [148] with an environment totally replete with oxygen, generating hyperoxic damage. Human milk has bioactive elements that protect newborns from cytotoxic damage to ROS during the first steps of life. These antioxidant properties of breast milk vary during the different stages of lactation being higher in colostrum and are linked to the maternal antioxidant condition, which could impact the antioxidant status of breast-fed infants [16]. Maternal nutrition influences the antioxidant concentrations in breast milk and consequently in the breastfed neonate [149].

Although it is a challenge to meet the optimal average of vitamins and mineral requirements for breastfed neonates, because the concentrations are highly variable, supplements with antioxidants in lactating mothers seem to increase the antioxidant property of human milk, protecting breastfed babies from infections and immunological diseases. It should be taken into account that there is a significant variation in vitamins content and a different susceptibility in individuals living in different regions, so it is difficult to recommend a specific dose [127]. Moreover, our study detected limitations regarding the relatively small number of participants, maternal dietary habits, milk sample analysis method, supplements’ doses and form of administration, which accentuated the differences among studies. Therefore, a balanced maternal diet continues to be essential to provide the complete transfer of antioxidants to the breastfed neonate, rather than from dietary supplements. Because a low antioxidant status of the mother may be transmitted to the infant at early unprotected stages of life, more studies about the effects of antioxidant supplements on neonatal health are required.

Although all of these results are favorable to the use of antioxidants, several aspects must be taken into account. First, commercial forms of certain antioxidants, such as RESV or EGCG, may vary in bioavailability and purity [150,151]. Previous studies in our laboratory determined the variation in bioavailability of EGCG depending on the mode of intake, obtaining greater bioavailability in the absence of additional food but also greater variability between individuals [43]. Therefore, it is desirable that clinical studies show a prior study about the pharmacokinetics of the antioxidant to determine the best form of administration and the real product purity. Second, it is necessary to use unified animal models. For example, in both PE and FGR studies, the authors used models obtained from intraperitoneal injection of compounds such as NG-Nitro-L-arginine methyl ester (L-NAME), desoxycorticosterone acetate (DOCA) [57,58,152] or by genetic knockout models [153]. This, together with the fact that antioxidant blood levels and their pharmacokinetics are not usually measured, can lead to inconclusive results [152]. Moreover, we have found great variability in the timing of antioxidant intake. Whether the effect of antioxidants may have a greater or lesser effect on these outcomes, depending on whether they are taken before or in the different trimesters of pregnancy is a question to be studied in depth. Additionally, the great variability in the studied populations, with different nutritional statuses according to the income level of the country of birth, make it difficult to obtain clear results.

A consistent interaction between maternal age and the effects of certain antioxidants has recently been demonstrated. Harville et al. observed that protective effects of antioxidants were largely limited to women older than 30, while negative effects predominated in younger women [89]. This study serves as a wake-up call on the need for age stratification in future studies on the effect of antioxidants during pregnancy.

Moreover, it is important to develop exhaustive safety studies before recommendations can be made in this at-risk population. Not all antioxidants of natural origin can be considered suitable for this population; certain antioxidants, such as crocin and safranal (active ingredients of saffron) have teratogenic effects in mice, despite their use for thousands of years in cooking [154]. This work has shown the therapeutic effect of certain antioxidants on the most prevalent diseases during pregnancy, but the safety of their long-term use needs to be deeply studied.

Finally, it is crucial to clarify how antioxidants may induce epigenetic modifications during pregnancy and thus reverse programmable diseases in order to improve the health outcomes of the neonate [155].

## Figures and Tables

**Table 1:** Antioxidants used in the treatment of preeclampsia and fetal growth restriction.

Antioxidant	Author (Year)/Country	Objectives	Study Design	Population	Gestational Age	Dose/Intervention Period	Variables Studied	Key Results	Conclusion	Quality of Evidence
Preeclampsia										
Curcumin	Fadinie et al. (2019) [28]/Indonesia	To determine the effect of perioperative curcumin administration on COX-2 and IL-10.	DBRCT	PEP undergoing CS. Age: 19–40 y PG (*n* = 23) TG (*n* = 24)	Full term.	100 mg/day 12 h before C-section.	COX-2 and IL-10 levels in serum at 90 min and 12 h.	NS differences.	Curcumin does not change COX-2 or IL-10 levels after preoperational administration.	Low ++/++++
EGCG	Shi et al. (2018) [33]/China	To determine the effect of coadministration of EGCG and nifedipine on PE.	DBRCT	Severe PEP Age: 25–35 y PG (*n* = 156) TG (*n* = 148)	~37 ± 1.5 w	100 mg/capsule up to 5 dosages (≥98% purity)/every 15 min until normalization of BP.	Time needed to control BP. Time to new hypertensive crisis. Number of treatment doses used.	Significantly less time to control blood pressure in TG vs. PG (31.2 ± 16.7 min vs. 45.3 ± 21.9 min) Time between hypertensive crises significantly longer in the TG group (7.2 ± 2.9 h vs. 4.1 ± 3.7 h). Use of EGCG decreased the number of doses required to control BP.	EGCG potentiates the efficacy of nifedipine against severe PE	High ++++/++++
Melatonin	Hobson et al. (2018) [34]/Australia	To evaluate the safety and efficacy of melatonin on PE clinical outcomes.	Open label, phase I single arm clinical trial.	20 women with preterm PE. 48 HCC	~32 ± 1 w	Melatonin tablets (10 mg) + vitamin B6 (10 mg)/3 times daily; from recruitment until delivery.	Maternal and perinatal safety. Prolongation of pregnancy. PE biomarkers.	Significant increase of the interval from diagnosis to delivery (6 ± 2.3 days) vs. HCC. NS differences in average mean arterial BP between groups. Melatonin significantly decreased antihypertensive medication. No disturbance in sleep patterns.	Melatonin is safe for newborns and their mothers and could provide effective adjuvant therapy to extend pregnancy duration.	Low ++/++++
Resveratrol	Ding et al. (2017) [35]/China	To determine the effect of coadministration of RESV and nifedipine on PE.	DBRCT	Severe PEP Age: 21–32 y PG (*n* = 175) TG (*n* = 174)	~34 ± 3.5 w	50 mg/capsule up to 5 dosages/ every 15 min until normalization of BP.	Time needed to control BP.Time to new hypertensive crisis. Number of treatment doses used.	Significantly less time to control blood pressure in TG vs. PG (35.6 ± 18.7 min vs. 51.1 ± 22.4 min). Time between hypertensive crises was significantly longer in the TG group (8 ± 2.1 h vs. 5.5 ± 1.8 h). Use of RESV significantly decreased the number of doses required to control BP.	RESV potentiates the efficacy of nifedipine against severe PE.	High ++++/++++
	Caldeira-Dias et al. (2019) [36]/Brasil	To study the effects of serum from PE women on antioxidant defenses and vasodilator factor in HUVECs cells.	Observational	Severe PEP (*n* = 6). Healthy women (*n* = 6).	~28 ± 4 w	No intervention, RESV was added to serum from PE and healthy patients.	ARE activation HO-1, GSH and NO levels Intracellular ROS levels.	NS differences in ARE activation in PE group. RESV decreased HO-1 levels by 15% in the PE group. RESV increased GSH and nitrite levels in the PE group. NS differences in ROS levels.	RESV could prevent alterations in HO-1 and NO markers in endothelial cells.	Very Low +/++++
Grape juice	Caldeira-Dias et al. (2021) [37]/Brasil	To compare theeffects of serum incubation in endothelial cells from PE women before and after 1 h of red grapefruit juice ingestion.	Pilot Phase I single-arm open-label clinical trial.	PEP (*n* = 4)	~25 ± 3 w	200 mL of organic whole grape juice.	Redox status and NOProduction.	Significant decrease of HO-1 and GSH levels (~17% and ~50%) in HUVEC cells compared to serum prior to juice ingestion. Significant decrease of ARE activity (~69%). Significant increase in NO levels. NS differences in ROS levels.	The biologically active molecules in grape juice restore the physiological NO balance of the endothelium.	Very Low +/++++
Fetal growth restriction										
Melatonin	Miller et al. (2014) [38]/Australia	To evaluate MLT as neuroprotectant.	Pilot Phase I single-arm open-label.	12 women with severe early onset IFGR. CG (*n* = 6) TG (*n* = 6)	~26 ± 1 w	MLT tablets (4 mg), twice daily, from recruitment until delivery.	Placental concentration of MDA.	Significantly higher levels of umbilical arterial MLT in TG vs. CG (6501 vs. 21 pg/mL). Significantly lower levels of MDA in the placenta in TG (2.4 vs. 4.6 nmol/mg tissue).	Antenatal MLT treatment reduces fetoplacental oxidative stress.	Low ++/++++

Abbreviations: ARE: antioxidant response element; BP: blood pressure; CG: control group; COX-2: cyclooxygenase-2; CS: cesarean section; DBRCT: double-blind, randomized clinical trial; EGCG: epigallocatechin gallate; GSH: glutathione; h: hours; HCC: historical comparative controls; HO-1: heme oxygenase-1; HUVEC: human umbilical vein endothelial cells; IFGR: intrauterine fetal growth restriction; IL-10: interleukin 10; MDA: malondialdehyde; min: minutes; MLT: melatonin; NO: nitric oxide; NS: no significant; PE: pre-eclampsia; PEP: preeclamptic patients; PG: placebo group; RESV: resveratrol; ROS: reactive oxygen species; TG: test group; w: weeks; y: years. Quality of evidence grades: high (++++), moderate (+++), low (++), very low (+).

**Table 2:** Antioxidant use in the prevention of prematurity.

Antioxidant	Author (Year)/Country	Objectives	Study Design	Population	Dose/Intervention Period	Variables Studied	Key Results	Conclusion	Quality of Evidence
Vitamin C	Martin et al. (2015), [85]/USA	To analyze the association between vitamin C intake and risk of PTB.	Prospective cohort study.	3143 pregnant women at 26–29 GWs.	FFQ for dietary information collection.	PTB before 37 GWs.	OR of PTB 1.55, 95% CI: 1.07–2.24 in pregnant women with high consumption of vitamin C-rich drinks.	Type of diet during pregnancy, including high consumption of vitamin C-rich products is associated with PTB.	Low ++/++++
	Ghomian et al. (2013) [86]/Iran	To study the association of vitamin C supplementation with the risk of PPROM in women with previous PPROM.	Clinical trial	170 singletons pregnancies at 14 GWs in women with previous PPROM. (85 controls/85 cases)	Cases: 100 mg vitamin C daily from 14 GWs. Controls: chewing a placebo tablet from 14 GWs.	Incidence of PPROM in pregnant women with history of previous PPROM.	PPROM in the control group 38 (44.7%) and 27 (31.8%) in the vitamin C supplementation group (*p* < 0.001).	Low vitamin C intake is associated with an increased risk of PPROM in women with history of previous PPROM.	Moderate +++/++++
	Gupta et al. (2020) [87]/India	To assess the association between vitamin C deficiency and PPROM.	Prospective case control study.	100 women aged 18–35 with singleton pregnancy between 28–36.6 GWs. (*n* = 50 PPROM/*n* = 50 control)	Blood test for ascorbic acid and IL-6 analysis.	Measurement of plasmatic ascorbic acid and Il-6 levels. PPROM before 37 GWs.	Plasmatic ascorbic acid levels in PPROM group 0.60 ± 0.35 and 1.18 ± 0.43 mg/dL in control group (*p* < 0.001). 33 patients (66%) with low plasmatic ascorbic acid levels in PPROM vs. 6 (12%) in control group (*p* < 0.001).	PPROM susceptibility is increased in pregnant women with vitamin C deficiency.	Low ++/++++
	Sharma et al. (2014) [88]/India	To evaluate the association between vitamin C and PPROM.	Prospective case control study.	40 pregnant women with singleton pregnancy between 28–37 GWs. (*n* = 20 controls/*n* = 20 PPROM cases)	Blood test for ascorbic assessment.	Maternal plasmatic ascorbic acid level analysis.	Plasmatic ascorbic acid in PPROM group 0.41 ± 0.08 vs. 0.84 ± 0.19 mg/dl in control group (*p* < 0.001) and inverse relationship were observed between PPROM relationship and ascorbic acid levels. A decrease in ascorbic acid levels was observed throughout pregnancy.	Antenatal vitamin C supplementation would prevent PPROM.	Low ++/++++
Vitamin E	Harville et al. (2020) [89]/USA	To check the relationship between preconceptionally antioxidant levels and obstetric adverse outcomes.	Prospective observational study.	2787 women aged 18–30 (1638 pregnant women during the follow-up period).	Blood test for antioxidant status assessment. Follow-up at 0, 2, 5, 7, 10, 15 and 20 years.	Interviewer-administered quantitative FFQ for evaluation of antioxidant intake. Plasmatic levels of the studied antioxidants. Women self-report pregnancy outcomes.	No statistically significant differences in vitamin E levels according to PTB.	Vitamin E supplementation was not associated with decrease in PTB.	Low ++/++++
	Bartfai et al. (2012) [90]/Hungary	To determine the effect of vitamin E in the prevention of PTB.	Observational study.	37,971 pregnant women (*n* = 35,864 no vitamin E/*n* = 2287 vitamin E).	Prospective record of maternal clinical history and obstetric outcomes. Retrospective maternal self-report about pregnancy supplements (estimated daily dose of 450 mg vitamin E).	PTB before 37 GWs.	Rate of PTB was 9.3% in women without vitamin E supplementation vs. 6.6% in vitamin E supplementation group (adjusted OR 0.71, 95% CI; 0.63–0.84).	Vitamin E was associated with a nearly 30% reduction in PTB.	Moderate +++/++++
	Carmichael et al. (2013) [91]/USA	To assess maternal dietary intake and PTB.	Transversal study.	5738 singleton pregnancies.	Shortened version FFQ.MDS and DQI for diet quality evaluation.	PTB before 37 GWs.	OR PTB < 32 GWs 1.9 (1.0–3.6) for the lowest quartile of vitamin E intake.	Vitamin E nutritional intake is not clearly associated with PTB.	Low ++/++++
Vitamin C + E	Gungorduk et al. (2014) [92]/Turkey	To study the effect of vitamin C + E supplementation on PPROM to increase the latency period before birth.	Prospective open randomized trial.	229 pregnant women with PPROM 24–34 GWs. (*n* = 126 1 g vitamin C + 400 IU vitamin E/*n* = 123 placebo)	Diagnosis of PPROM according clinical examination, nitrazine test or Amnisure^®^ test.	Latency period until birth.	Longer latency period before birth in vitamin C + E group (11.2 ± 6.3 days) compared with control group (6.2 ± 4.0 days), *p* < 0.001; and higher gestational age (31.9 ± 2.6 weeks vs. 31.0 ± 2.6 weeks), *p* < 0.01.	Vitamin C + E supplementation is associated with a longer latency period before birth and higher gestational age at birth.	Moderate +++/+++++
	Hassanzadeh et al. (2016) [93]/Iran	To evaluate the relationship between macro and micronutrients maternal intake in 3rd trimester and PPROM.	Prospective cohort study.	620 pregnant women aged 15–49 years.	48-h dietary recalls at 11th–15th, 26th, 34th–37th GWs. Records of physical activity and reproductive and demographic maternal characteristics.	PPROM diagnosis.	Vitamin C levels in 1st and 2nd trimester were higher in PPROM (206.2 ± 156.5 and 208.7 ± 193.1) compared with controls (147.9 ± 99.8 and 152.7 ± 105.8), *p* = 0.020 and *p* = 0.037. No statistically significant differences in vitamin E levels.	Higher vitamin C intake in 1st and 2nd trimester of pregnancy was associated with an increased risk of PPROM.	Low ++/++++
	Hauth et al. (2010) [94]/USA	To assess the protective effect of vitamin C and E supplementation in PTB prevention.	Randomized, double-masked, placebo-controlled trial.	10,154 nulliparous women with low-risk pregnancies (*n* = 4992 1000 mg vitamin C + 400 IU vitamin E/*n* = 4976 placebo).	Administration of 1000 mg ascorbic acid + 400 IU α-tocopherol acetate or placebo (mineral oil) since 9.0–16.6 GWs to birth.	Spontaneous PTB and PROM + PTB.	PROM + PTB before 32 GWs in the supplemented pregnant women (0.3%) compared to the placebo group (0.6%), adjusted OR 0.3–0.9. PPROM < 32 GWs 0.36% in supplement group vs. 0.64% in the placebo group, *p* < 0.046.	Maternal supplementation with vitamin C + E in low-risk pregnancies does not reduce total spontaneous PTB, but prevent PROM + PTB before 32 GWs.	High ++++/++++
	Ilhan et al. (2015) [15]/Turkey	To analyze maternal oxidative status in PPROM.	Prospective cross-sectional study.	72 pregnant women. (*n* = 38 PPROM/*n* = 34 controls)	Blood samples collection. ELISA for biomarker analysis.	Plasmatic IL-6, vitamin C, vitamin E, CRP and 8-isoprostane levels, TOS and TAS.	High TOS and low vitamin C and 8-isoprostane in PPROM group (*p* < 0.001).	Plasmatic vitamin C levels were associated with PPROM.	Low ++/++++
	Ilhan et al. (2017) [95]/Turkey	To investigate maternal oxidative status in PPROM and the latency period to birth.	Prospective case control study.	116 pregnant women. (*n* = 75 PPROM cases between 24–34 GWs/*n* = 41 controls)	Maternal blood for biochemical analysis. ELISA test. HPLC analysis.	Plasmatic vitamin C, vitamin E, MDA, leukocyte count and CRP levels. TOS and TAS evaluations.Latency period between PPROM and birth.	Vitamin C levels were lower in PPROM group (7.39 ± 2.37) compared to controls (13.83 ± 3.16), *p* < 0.001, but not statistically significant differences were found in vitamin E levels.	Vitamin C is associated with a lower risk of PPROM.	Low ++/++++
	Koenig et al. (2017) [96]/USA	To assess the relationship between nutrient intake and cervix characteristics.	Longitudinal descriptive design.	47 pregnant women.	FFQ at 19–24, 27–29 GWs. Transvaginal ultrasound examination at 19–21, 23–25, 27–29, 31–33 and 35–37 GWs.	Cervical remodeling.	Women in the less-risk group of PTB assessed by cervical length remodeling had higher vitamin E intake (*p* = 0.04). No differences according to vitamin C intake.	Certain nutrients, such as vitamin E, prevented PTB through the inhibition of premature cervical remodeling.	Very low +/++++
	Zhang et al. (2017) [97]/China	To evaluate the association between dietary nutrients and PTB.	Prospective case-control design.	511 pregnant women. (*n* = 130 PTB/*n* = 381 term birth)	FFQ for mother diet assessment.	PTB before 37 GWs.	Women with PTB had lower vitamin E intake (29.60 ± 9.51) than women with term birth (33.57 ± 11.30), *p* < 0.01. Women with PTB and BMI <18.5 had lower vitamin E intake (*p* < 0.05). No statistically significant differences in vitamin C intake and PTB.	Low levels of vitamin E intake were associated with PTB.	Low ++/++++
Zinc	Charkamyani et al. (2019) [98]/Iran	To study the effect of a diet modification program in IVF pregnant women to reduce PTB.	Quasi-experimental clinical trial.	170 IVF pregnant women aged 19–45 from 2017 to 2018.	Dietary intervention promoting increased intake of lactose, fiber, magnesium, zinc, vitamin B3 and B5.	Self-developed questionnaire for demographic characteristics collection, dietary habits and lifestyle behaviors.	Zn increased intake (*p* = 0.017) after dietary intervention. No statistically significant differences in the rate of PTB according to Zn intake.	Zn intake is not associated with PTB in IVF pregnant women.	Low ++/++++
	Nga et al. (2020) [99]/Vietnam	To determine if nutrient-rich diet during pregnancy improves obstetric outcomes in low-income countries.	Randomized controlled trial.	460 primiparous women aged 18–30 from 2011 to 2015. (*n* = 150 PC-T, *n* = 153 MG-T, *n* = 157 RPC)	3 study groups: PC-T; MG-T and RPC. Nutrient-rich food-based supplement containing Zn, folate, vitamin B12, A and iron.	Zn intakes increase in PC-T and MG-T groups (*p* < 0.001) compared to RPC.	No statistical differences in PTB according to the intervention group.	A nutrient-rich supplement containing Zn in pregnant women from low-income countries did not improve the rate of PTB.	Moderate +++/++++
	Nossier et al. (2015) [100]/Egypt	To assess the effects of Zn supplementation on obstetric outcomes.	Double-blind randomized controlled trial.	1460 women with low serum Zn levels from 2007 to 2009 (*n* = 223 placebo, *n* = 225 Zn, *n* = 227 Zn + MM)	3 study groups: (1) placebo; (2) Zn (daily 30 mg ZnSO_4_); (3) Zn + MM (daily 30 mg ZnSO_4_ + multivitamin).	FFQ for dietary intake assessment. Blood test for serum Zn quantification.	Higher Zn serum levels in the Zn group compared to placebo and Zn + MM (*p* < 0.001). PTB was lower in the Zn group (1%) and Zn + MM group (2%), compared to placebo (10%), *p* < 0.001. RR = 0.012, 95% CI 0.036–0.77) in the Zn group and RR = 0.268, 95% CI 0.119–0.603 in Zn + MM group compared to the placebo group.	Zn supplementation is effective in reducing PTB.	High ++++/++++
	Zahiri et al. (2015) [101]/Iran	To investigate the effect of Zn supplementation on obstetric outcomes.	Randomized controlled trial.	540 women from 2010–2012 (*n* = 270 Zn/*n* = 270 control).	2 study groups (supplementation from 16 GWs until delivery): (1) daily supplementation 400 µg folic acid + 30 mg ferrous sulfate; (2) daily supplementation 400 µg folic acid + 30 mg ferrous sulfate + 15 mg Zn sulfate.	Demographic and anthropometric data, blood pressure and obstetric outcomes.	No statistically significant differences in PTB (*p* = 0.999) or PPROM (*p* = 0.630).	Daily 15 mg Zn supplementation does not reduce PTB or PPROM.	High ++++/++++
	Costa et al. (2018) [102]/Portugal	To examine obstetric outcomes in women who had undergone bariatric surgery.	Retrospective descriptive observational study.	39 pregnant women.	Pregnant women who had previously undergone bariatric surgery. Study period 2010–2014.	Maternal characteristics, type of bariatric surgery (restrictive or mixed technique), obstetric outcomes. Evaluation of Zn, iron, vitamin B12 and D prior and during pregnancy.	Zn deficiency in 12 cases (66.8%). PPROM in 2 cases. PTB in 5 cases.	No differences in obstetric outcomes were observed. Nutritional deficits are less common in restrictive bariatric surgery.	Very low +/++++
	Kucukaydin et al. (2018) [103]/Turkey	To analyze trace element, heavy metals, and maternal vitamin in PTB and PPROM.	Prospective cohort study.	68 women with PTB (*n* = 35 PPROM, *n* = 33 without PPROM).	Singleton pregnancies. Study period: 2008–2009.	Zn levels in maternal, umbilical plasma and placental tissue.	Zn lower levels in maternal and umbilical cord serum in PPROM (*p* < 0.01). No statistically significant differences in placental tissue Zn levels.	PPROM is associated with low maternal and fetal Zn levels.	Low ++/++++
	Shen et al. (2015) [104]/China	To evaluate changes in trace elements during pregnancy and related-obstetric outcomes.	Prospective cohort study.	1568 pregnant women.	Recruitment of women aged 18–39 in antenatal care.Study period 2013–2014. Blood tests in fasting conditions.	Measurement of plasmatic Zn levels before pregnancy, at 7–12 GWs, 24–28 GWs and 35–40 GWs.	No statistically significant differences in Zn levels during pregnancy. Zn levels were significantly lower in PPROM and PTB (*p* < 0.05) compared to controls.	Zn deficiencies in pregnancy may be associated with increased risk of PPROM and PTB.	Low ++/++++
Tea	Chen et al. (2018) [105]/Ireland	To evaluate the association between caffeine intake (tea) and birth outcomes.	Prospective cohort study.	941 mother-child dyads	FFQ to measure maternal tea intake during the first 12–16 weeks of pregnancy.	Average tea consumption frequency (grams per day) divided in 6 levels. PTB (before 37 weeks of gestation).	PTB (OR = 2.56 (1.14–5.75)) in highest tea intake categories compared to the lowest (*p* < 0.05).	Maternal tea drinking is associated with an increased risk of PTB.	Low ++/++++
	Huang et al. (2016) [106]/China	To study the relation between tea consumption and risk of PTB.	Prospective cohort study	10,179 women with uncomplicated pregnancies.	Standardized and structured questionnaires within 3 days after labor to obtain information regarding tea consumption.	Amount of tea consumption. PTB: moderate PTB (32–36 weeks), very PTB (28–31 weeks) and extremely PTB (<28 weeks).	PTB (OR = 1.36, 95% CI: 1.09–1.69) in tea consumers. Moderate PTB (OR = 1.41, 95% CI: 1.12–1.79) and spontaneous PTB (OR = 1.41, 95% CI: 1.09–1.83) in tea consumers.	Tea intake (green and scented tea) during pregnancy is associated with PTB.	Low ++/++++
	Lu et al. (2017) [107]/China	To assess the association between tea consumption in early pregnancy and risk of PTB.	Prospective cohort study.	8775 pregnant women.	Self-completed questionnaire about sociodemographic variables and tea drinking at 16 weeks.	Amount of tea consumption and type of tea.PTB (before 37 weeks of gestation).	No statistically significant differences in PTB according to the amount and type of tea.	Tea drinking in early pregnancy is not associated to increased risk of PTB.	Low ++/++++
	Okubo et al. (2015) [108]/Japan	To examine the association between caffeine intake with the risk of PTB.	Prospective cohort study.	858 mother-child dyads.	Validated self-administered dietary history questionnaire (8 categories) collected through gestation.	Maternal total caffeine intake. PTB (before 37 weeks gestational age).	Maternal median caffeine intake = 258 mg/day. Tea drinking (1 cup/day) is associated with an increased risk of PTB (OR 1.16; 95% CI 1.01–1.32, *p* = 0.035).No differences in risk of PTB according to the trimester of caffeine intake.	Tea consumption is associated with an increased risk of PTB.	Low ++/++++
	Moussally et al. (2010) [109]/Canada	To analyze the association between HP consumption (mainly green tea) and PTB.	Prospective cohort study.	8505 pregnant women aged 15–45 years.	Self-administered questionnaire in the second or third trimester of pregnancy.	Consumption of HP (green tea) during pregnancy. PTB (before 37 weeks gestational age)	No association between green tea intake and risk of PTB (OR 0.94 (0.55–1.61)).	Green tea drinking in the second and third trimester of pregnancy is not associated with an increased risk of PTB.	Low ++/++++
	Sengpiel et al. (2013) [110]/Norway	To investigate the association between maternal caffeine consumption and birth results.	Prospective cohort study.	59,123 mother-child dyads.	Self-administered FFQ at 17, 22 and 30 weeks of pregnancy.	Caffeine calculation using FoodCalc and Norwegian Food Composition table. Spontaneous PTB (22–36 weeks of gestation).	Black tea was associated with elevated risk of early PTB (OR 1.61, 95% CI 1.10–2.35, *p* = 0.01), but not an association in all PTB. The other sources of caffeine were not associated with an increased risk of PTB.	Black tea is associated with the risk of PTB. Caffeine intake from other sources (coffee, caffeinated soft drinks, tea and chocolate) is not associated with PTB.	Low ++/++++
	Sindiani et al. (2020) [111]/Jordan	To study the association between tea consumption and PTB.	Unmatched case-control study.	1110 healthy pregnant women. (314 cases/796 controls)	Interviewer administered structured questionnaires in women admitted for delivery.	Average number of teacups (150 mL) consumption. PTB (before 37 weeks gestational age).	Tea drinking was not associated with elevated risk of PTB.	There is not an association between tea consumption and the risk of PTB.	Very low +/++++
Melatonin	Biran et al. (2019) [112]/France	To compare melatonin plasmatic levels in women who delivered preterm infants before 34 GW.	Prospective longitudinal multicenter study.	169 mothers.	Recruitment of women admitted for birth and blood tests for analysis of plasma melatonin levels.	Radioimmunoassay for plasma melatonin concentration measurements.	Statistically significant lower median IQR = 7 (7–20) in mothers who delivered before 34 GW compared to median IQR 11 (7–50) in mothers who gave birth after 34 weeks (*p* = 0.02).	Median plasma melatonin concentration was significant lower in mothers who delivered before 34 gestational weeks.	Low ++/++++
	Dominguez et al. (2014) [113]/Argentina	To evaluate the effect of melatonin treatment in a mice model of inflammation-associated PTB.	BALB mice model.	4 experimental groups (*n* = 10 each group): (1) control; (2) melatonin pellet; (3) LPS injection; (4) melatonin + LPS.	Sc administration of 25 mg melatonin pellet on GD 14. Ip injection of bacterial LPS twice/day on GD 15.	Gestational age at the moment of the birth. NOS activity assessment. PG radioimmunoassay. ELISA for TNFα measurement. Western blot for iNOS and COX-2 activity analysis.	Melatonin prevented 50% of LPS-induced PTB (*p* > 0.05). PGE2, COX-2, PGF2α levels, NOS and iNOS activity were decreased in LPS + melatonin compared to the LPS group (*p* < 0.05).	Melatonin has effect on inflammation-induced alterations, making it a promising agent for PTB prevention.	Very low +/++++
	Lee et al. (2019) [114]/Korea	To study the immunomodulatory effect of melatonin on PTB in a murine model.	Mouse model and in vitro model.	3 experimental groups (*n* = 10 for each group): (1) control; (2) LPS; (3) LPS + melatonin.	2 mg/Kg LPS ip injection on 16.5 GD. 10 mg/Kg melatonin ip injection on 16.5 GD (30 min after LPS injection).	Gestational age at birth. Western blot analysis for SIRT1/Nrf2 analysis. RT-PCR for IL-1β, IL-6, TNF-α, COX-2 quantification.	Melatonin decreases a 30% the rate of PTB (*p* < 0.001). Melatonin significantly decreased TNF-α, COX-2, IL-6 and 1L-1β levels (*p* < 0.05) and increased SIRT1 and Nrf2 levels (*p* < 0.05).	The effect of melatonin in the reduction of PTB is related to its immunomodulatory effects.	Very low +/++++
	Ramiro-Cortijo et al. (2020) [115]/Spain	To investigate the effect of melatonin on PTB in twin pregnancies.	Single-center prospective observational study.	104 twin-pregnant women.	Blood test between 9–11th GW.	Spectrophotometry for antioxidant (catalase, SOSA, GSH, thiol groups, phenolic compounds) and oxidative damage biomarkers (MDA, carboxyl groups) analysis and assessment of global antioxidant status (Antiox-S, Prooxy-S). Immunoassay for melatonin quantification.	Melatonin was significant lower in women with PTB (*p* = 0.024) compared to full-term. No differences in Antiox-S and Prooxy-S according to PTB.	Lower melatonin levels in the first trimester were associated with PTB in twin pregnancies.	Very low +/++++
	Specht et al. (2019) [116]/Denmark	To evaluate the relationship between night work during first and second trimesters of pregnancy and risk of PTB.	Prospective cohort study.	16,501 pregnant women.	Pregnant women with nightshift (23:00–06:00) in their first (1–12 GW) or second trimester (13–22 GW) from 2007 to 2013.	Odds of PTB (23–37 GW) analysis.	Prevalence of PTB was 5.2% in night workers and 5.1% in day workers.	There was no association between the night working shift and the risk of PTB.	Low ++/++++

Abbreviations: BMI: body mass index; COX-2: cyclooxygenase 2; CRP: C-reactive protein; DQI: Diet Quality Index for pregnancy; ELISA: enzyme-linked immunosorbent assay; FFQ: food frequency questionnaire; GD: gestational day; GSH: reduced glutathione; GWs: gestational weeks; HP: herbal products; HPLC: high-performance liquid chromatography; IL: interleukin; iNOS: inducible nitric oxide synthase; Ip: intraperitoneal; IQR: interquartile range; IVF: in vitro fecundation; LPS: lipopolysaccharide; MDA: malondialdehyde; MDS: Mediterranean Diet Score; MG-T: mid gestation supplementation to term; MM: multivitamins; NOS: nitric oxide synthase; Nrf2: nuclear factor-erythroid 2-related factor 2; OR: odds ratio; PC-T: preconception to term supplementation; PG: prostaglandin; PGE2: prostaglandin E2; PGFα: prostaglandin Fα; PPROM: preterm premature rupture of membranes; PROM: premature rupture of membranes; PTB: preterm birth; RR: relative risk; SIRT: silent information regulator factor transcript-1; RPC: routine prenatal care; RT-PCR: reverse transcription polymerase chain reaction; SOSA: superoxide anion scavenging activity; TAS: total antioxidant status; TNFα: tumor necrosis factor α; Sc: subcutaneous; TOS: total oxidative status; Zn: zinc. Quality of evidence grades: high (++++), moderate (+++), low (++), very low (+).

**Table 3:** Clinical trials about antioxidants supplements in lactating mothers.

Antioxidant	Author (Year)/Country	Objectives	Study Design	Population	Dose/Intervention Period	Variables Studied	Key Results	Conclusion	Quality of Evidence
Vitamin C	Hoppu et al. (2005) [120]/Finland	To evaluate the impact of vitamin C in breast milk on the development of atopic disease.	Cross-sectional.	65 mothers with atopic background at the end of gestation and their infants.	Mother’s diet rich in natural supplies of vitamin C (abundant intake of fresh fruits, berries and vegetables during breastfeeding).	Concentration of antioxidants in breast milk Infants: Clinical atopy and SPT at 12 months.	Decreased risk of atopy in the infant (OR = 0.30; 95% CI 0.09–0.94; *p* = 0.038).	A maternal diet enriched in natural supply of vitamin C during breastfeeding may decrease the risk of atopy in high-risk infants.	Low ++/++++
Vitamin C and E	Zarban et al. (2015) [121]/Iran	To examine the effects of vitamin C and E supplements in the diet of breastfeeding mothers to ameliorate antioxidant activity.	RCCT	Breastfeeding mothers. CG: 30 EG: 30	CG: free diet. EG: free diet supplemented with effervescent tablets of vitamin C (500 mg) and chewable tablets of vitamin E (100 IU).	Antioxidant content and activity in breast milk and infants’ urine, respectively. Measurements: the ferric reducing/antioxidant properties.	EG: higher levels of antioxidants in the breast milk (610–295.5 to 716–237.5 μmol/L) and infant urine (43.2–21.8 to 75.0–49.2 μmol/mg creatinine) (*p* < 0.05). Free radical scavenging in infant urine after 30 days of supplementation (*p* < 0.05).	Supplements of vitamin C and E increase anti-oxidant content of breast milk and antioxidant activity in infant urine.	High ++++/++++
Vitamin C	Friel et al. (2007) [123]/Canada	To determine if iron or iron + vitamin C or iron + TVS supplementation (vitamins A, C and D) improve lipid oxidation in human milk in vitro.	Experimental.	81 mothers. Preterm babies: 29–37 weeks. HM samples.	Iron = 2 mg/kg/day. Vitamin C = 20 mg/kg/day.	Lipid peroxidation in HM (FOX-2 and TBARS assays). Fatty acid composition (gas chromatography). Intracellular oxidative stress or DNA damage (cell culture bioassays: Caco-2BBe and FHS-74 Int cells).	Iron; iron + vitamin C; iron + TVS: ↑ lipid oxidation products of HM ↓ mono and polyunsaturated fatty acids in HM. Iron; iron + TVS: ↑ intracellular oxidative stress in FHS-74 Int cells. All treatments increased DNA damage in Caco-2BBE cells.	Iron + vitamin C increased DNA damage if compared to iron alone.Iron supplements may provoke oxidative stress in preterm infants and should be divided from vitamin C supplementation.	Very Low +/++++
	Daneel-Otterbech et al. (2005) [122]/Canada	To compare human milk AA content in European and African women and to evaluate the influence of increased AA intake on human milk AA output.	RCCT	171 African lactating women. 142 European women.	Effervescent tablets (1000 mg AA/day).	AA concentration in human milk.	After 10 d: ↑ AA concentration from 19 to 60 mg/kg (*p* ≤ 0.001) and from 60 to 70 mg/kg (*p* ≤ 0.03) in 18 African and 10 European women. In 11 African women, AA levels increased from 17 to 36 mg/kg (*p* ≤ 0.001) after intake of 100 mg AA/day during the same period.	AA in human milk can be increased in women with low human milk AA content at baseline	Moderate +++/++++
Vitamin E	Melo et al. (2017) [125]/Brasil	To evaluate if supplementation with vitamin E increases the concentration of α-TOH in colostrum and its supply to the newborn.	RCCT	*n* = 99 healthy adult pregnant women. (CG: 39; EG: 60)	The supplemented group received 400 IU of supplementary vitamin E.	Vitamin E concentrations in human milk and blood sample, before and after treatment.	Basal vitamin E levels: CG: 1509.3 ± 793.7 g/dL EG: and 1452.9 ± 808.6 g/dL After 24 h: CG: 1650.6 ± 968.7 g/dL (*p* > 0.05) EG: 2346.9 ± 1203.2 g/dL (*p* < 0.001) ↑ of vitamin E in the newborn to 9.3 mg/day.	Maternal vitamin E supplementation provides more than twice the Recommended Daily Intake of this vitamin.	High ++++/++++
	Medeiros et al. (2016) [126]/Brasil	To evaluate the effect of maternal vitamin E supplements on its levels in the colostrum, transitional milk and mature milk of mothers of preterm babies.	RCCT	*n* = 89 women (CG:51;EG:38)	400 IU of RRR-α-tocopheryl acetate. Breast milk samples were collected 1, 7 and 30 d after delivery.	Vitamin E concentrations in HM and BS by HPLC.	No significant differences in α-TOH levels in BS at baseline in both groups.Breast milk α-TOH levels increased by 60% at 24 h in EG. Transitional milk’s levels were 35% higher in EG. Similar α-TOH in the mature milk in both groups	Maternal supplements with 400 IU of RRR-α-tocopherol increased the vitamin E levels in the colostrum and transitional milk, but not of the mature milk. The effects of megadoses are not prolonged.	High ++++/++++
	Clemente et al. (2015) [135]/Brasil	To assess if supplements with a natural or synthetic form of α-TOH to lactating women increase its concentration in colostrum.	RCCT	*n* = 109 lactating women: CG: 36 NF:40 SF:33	Blood and colostrum samples were collected before and after supplementation to check the nutritional status of these women.	Vitamin E concentrations in HM and BS by HPLC.	Higher levels of α-TOH in colostrum from women who received supplementation (increase of 57% and 39% in NF and SF, respectively)	Supplements of α-TOH increase vitamin E concentrations in colostrum. However, the natural form is more efficient in increasing levels.	High ++++/++++
Selenium and zinc	Strambi et al. (2004) [129]/Italy	To compare the nutritional Se status in the AGA and SGA newborns in the first month of life in relation to feeding type.	Longitudinal	*n* = 210 (AGA: 129; SGA: 81) Feeding type: breast milk, formula and mixed.	Breast, bottle, or mixed feeding during the study period/4 weeks.	Se status in plasma and erythrocyte concentrations.	Se plasmatic levels were lower in SGA than in AGA newborns. SGA: higher plasma concentrations in breast-fed (*p* = 0.013) and mixed-fed (*p* = 0.006). The difference was not significant in AGA neonates.	Breast-fed SGA newborns showed higher plasma Se concentrations than formula-fed newborns. Even if supplemented from birth, Se intake was not adequate in bottle-fed SGA infants.	Moderate +++/++++
	Loui et al. (2004) [131]/Germany	To assess mineral, trace element, thyroid status and growth of infants fed with HM fortified with calcium, phosphorus and protein, with (BMF) or without (FM 85) trace elements (zinc, copper, manganese and iodine).	RCCT	*n* = 62 (FM85:34; BMF:28) Age: <33 w Weight: 1000–1499 g	Fortified HM with trace elements (5% BMF) or without (3% FM85)/6 weeks.	Serum: red blood cells. HM: minerals and trace elements. Serum: alkaline phosphatase activity, TSH, T4. FT4 on the fifth day and at 3 and 6 weeks of life. Clinical evolution and anthropometric measurements.	Levels of zinc, copper, manganese, calcium, phosphorus and magnesium were higher in the BMF group (*p* < 0.001). Serum zinc concentrations did not differ between groups. Median alkaline phosphatase activity: 436/379 IU/L in the FM 85/BMF group at 6 weeks (*p* < 0.01). Significant higher weight gain in the FM 85 group (due to higher caloric and protein intake) at 3 weeks.	zinc statusdid not differ between groups after treatment.	High ++++/++++
	Shaaban et al. (2005) [130]/Egypt	To assess the impact of maternal Zinc supplements on maternal and infant Zn levels and on the infants’ physical growth.	RCCT	60 primiparous lactating mothers.(CG:30; EG:30)	10 mg/day of Zinc sulfate capsules/2 months.	Zn levels in hair, nails and breast milk.	Zn supplements increased maternal Zn store in hair, nail, and breast milk. No differences in infant growth.	Zn supplements in lactating women increased breast milk Zn levels and maternal body stores, but it does not impact the infants’ physical growth.	High ++++/++++
Melatonin	Qin et al. (2019) [133]/China	To assess the changes in breast milk melatonin during lactation and to explore changes in melatonin levels and rhythms in preterm and term breast milk.	Longitudinal.	392 breast milk samples from 98 healthy nursing mothers at 0 to 30 days postpartum. 32% preterm. 67% full-term.	Breast milk was collected sequentially the same day, at 03:00, 09:00, 15:00.	Melatonin concentration.	Preterm and term breast milk: melatonin showed a circadian rhythm with peak at around 03:00. Highest melatonin concentration in the colostrum. Higher concentrations of melatonin in preterm than in term breast milk in the colostrum (28.67 pg/mL vs. 25.31 pg/mL, *p* < 0.022), transitional breast milk (24.70 pg/mL vs. 22.55 pg/mL), and mature breast milk (22.37 pg/mL vs. 20.12 pg /mL).	Melatonin showed a clear circadian rhythm in both preterm and term breast milk during lactation stages. The peak level was highest in colostrum, decreasing during the first month after birth.	Moderate +++/++++
	Honorio-Franca et al. (2013) [134]/Brasil	To assess the effects of HM samples (diurnal/ nocturnal) on colostral melatonin levels and the property of this hormone to modify colostral phagocyte activity.	Experimental.	60 Colostrum samples from 30 mothers during the day and night.	Not applicable.	Melatonin levels in colostrum and superoxide release and bacterial killing by colostral phagocytes.	Nocturnal colostrum: higher melatonin levels and increased spontaneous superoxide release; higher phagocytosis rate. Bactericidal activity of mononuclear phagocytes increased in response to melatonin, regardless of the sample type.	Melatonin levels in human colostrum follow a day-night cycle and increase phagocytic activity of colostral cells against bacteria.	Very low +/++++

Abbreviations: AA: ascorbic acid; AGA: adequate for gestational age; BS: blood sample; CG: control group; EG: experimental group; FHS-74: Human fetal small intestine cells; FOX-2: Ferrous ion oxidation xylenol orange-2; HM: Human milk. HPLC: high-performance liquid chromatography; IU: International Unit; NF: natural form; RCCT: randomized concentration-controlled trial; Se: selenium; SF: synthetic form; SGA: small for gestational age; SPT: skin prick test; TBARS: Thiobarbituric acid reactive substance; TSH: thyroid stimulating hormone; TVS: Trivisol; T4: thyroxine; Zn: Zinc. α-TOH: alpha-tocopherol; ↓: decrease; ↑: increase. Quality of evidence grades: high (++++), moderate (+++), low (++), very low (+).

## Supplementary Materials

The following are available online at https://www.mdpi.com/article/10.3390/antiox11040648/s1. Table S1: Grading of Recommendations Assessment, Development, and Evaluation (GRADE) of selected studies.

## Abbreviations

AGA	Appropriate for gestational age
Akt	Protein kinase B
ARE	Antioxidant Response Element
COX-2	Cyclooxygenase-2
EGCG	Epigallocatechin-3-gallate
FGR	Fetal growth restriction
GD	Gestational day
GRAS	Generally recognized as a safe
GSH	Glutathion
HMGB1	High mobility group box 1
HO-1	Heme oxygenase-1
HUVECs	Human umbilical vein endothelial cells
LP	Low-protein
LPS	Lipopolysaccharides
MCP-1	Monocyte chemoattractant protein-1
MDA	Malondialdehyde
NF-κB	Nuclear Factor κB
NO	Nitric oxide
NRF2	NFE2-related factor-2
PE	Preeclampsia
PLGF	Placental growth factor
PPROM	Preterm premature rupture of membranes
PTB	Preterm birth
RCT	Randomized controlled trial
RESV	Resveratrol
ROS	Reactive oxygen species
RUPP	Reduced uterine perfusion pressure
SGA	Small for gestational age
SIRT-1	Sirtuin-1
sPTB	Spontaneous PTB
TGF-β	Transforming growth factor-beta
TLR4	Toll Like Receptor 4
TNF-α	Tumor necrosis factor-alpha.
WHO	World health organization

## References

[1] Duley L. (2009). The Global Impact of Pre-eclampsia and Eclampsia. Semin. Perinatol..

[2] Steegers E.A.P., Von Dadelszen P., Duvekot J.J., Pijnenborg R. (2010). Pre-eclampsia. Lancet.

[3] Ødegård R.A., Vatten L.J., Nilsen S.T., Salvesen K.Å., Austgulen R. (2000). Preeclampsia and fetal growth. Obstet. Gynecol..

[4] Suhag A., Berghella V. (2013). Intrauterine Growth Restriction (IUGR): Etiology and Diagnosis. Curr. Obstet. Gynecol. Rep..

[5] Fall C.H.D. (2013). Fetal programming and the risk of noncommunicable disease. Indian J. Pediatr..

[6] Garite T.J., Clark R., Thorp J.A. (2004). Intrauterine growth restriction increases morbidity and mortality among premature neonates. Am. J. Obstet. Gynecol..

[7] Blencowe H., Cousens S., Oestergaard M.Z., Chou D., Moller A.B., Narwal R., Adler A., Vera Garcia C., Rohde S., Say L. (2012). National, regional, and worldwide estimates of preterm birth rates in the year 2010 with time trends since 1990 for selected countries: A systematic analysis and implications. Lancet.

[8] Walani S.R. (2020). Global burden of preterm birth. Int. J. Gynecol. Obstet..

[9] Cave C., Hanson C., Schumacher M., Lyden E., Furtado J., Obaro S., Delair S., Kocmich N., Rezac A., Izevbigie N.I. (2018). A comparison of vitamin E status and associated pregnancy outcomes in maternal–infant dyads between a Nigerian and a United States population. Nutrients.

[10] Haas D.M. (2011). Preterm birth. BMJ Clin. Evid..

[11] Hansson S.R., Nääv Å., Erlandsson L. (2015). Oxidative stress in preeclampsia and the role of free fetal hemoglobin. Front. Physiol..

[12] Guvendag Guven E.S., Karcaaltincaba D., Kandemir O., Kiykac S., Mentese A. (2013). Cord blood oxidative stress markers correlate with umbilical artery pulsatility in fetal growth restriction. J. Matern. Neonatal Med..

[13] Biri A., Bozkurt N., Turp A., Kavutcu M., Himmetoglu Ö., Durak I. (2007). Role of oxidative stress in intrauterine growth restriction. Gynecol. Obstet. Investig..

[14] Takagi Y., Nikaido T., Toki T., Kita N., Kanai M., Ashida T., Ohira S., Konishi I. (2004). Levels of oxidative stress and redox-related molecules in the placenta in preeclampsia and fetal growth restriction. Virchows Arch..

[15] Ilhan N., Celik E., Kumbak B. (2015). Maternal plasma levels of interleukin-6, C-reactive protein, vitamins C, E and A, 8-isoprostane and oxidative status in women with preterm premature rupture of membranes. J. Matern. Neonatal Med..

[16] Zarban A., Taheri F., Chahkandi T., Sharifzadeh G., Khorashadizadeh M. (2009). Antioxidant and radical scavenging activity of human colostrum, transitional and mature milk. J. Clin. Biochem. Nutr..

[17] Yuksel S., Yigit A.A., Cinar M., Atmaca N., Onaran Y. (2015). Oxidant and antioxidant status of human breast milk during lactation period. Dairy Sci. Technol..

[18] Shamseer L., Moher D., Clarke M., Ghersi D., Liberati A., Petticrew M., Shekelle P., Stewart L.A., Altman D.G., Booth A. (2015). Preferred reporting items for systematic review and meta-analysis protocols (prisma-p) 2015: Elaboration and explanation. BMJ.

[19] Liberati A., Altman D.G., Tetzlaff J., Mulrow C., Gøtzsche P.C., Ioannidis J.P., Clarke M., Devereaux P.J., Kleijnen J., Moher D. (2009). The PRISMA statement for reporting systematic reviews and meta-analyses of studies that evaluate health care interventions: Explanation and elaboration. J. Clin. Epidemiol..

[20] Page M.J., McKenzie J.E., Bossuyt P., Boutron I., Hoffmann T.C., Mulrow C.D., Shamseer L., Tetzlaff J.M., Akl E., Brennan S.E. (2021). The prisma 2020 statement: An updated guideline for reporting systematic reviews. BMJ.

[21] Guyatt G., Oxman A.D., Akl E.A., Kunz R., Vist G., Brozek J., Norris S., Falck-Ytter Y., Glasziou P., Debeer H. (2011). GRADE guidelines: 1. Introduction-GRADE evidence profiles and summary of findings tables. J. Clin. Epidemiol..

[22] Hooijmans C.R., De Vries R.B.M., Ritskes-Hoitinga M., Rovers M.M., Leeflang M.M., IntHout J., Wever K.E., Hooft L., de Beer H., Kuijpers T. (2018). Facilitating healthcare decisions by assessing the certainty in the evidence from preclinical animal studies. PLoS ONE.

[23] Nanda S., Madan K. (2021). The role of Safranal and saffron stigma extracts in oxidative stress, diseases and photoaging: A systematic review. Heliyon.

[24] Tabrizi R., Vakili S., Akbari M., Mirhosseini N., Lankarani K.B., Rahimi M., Mobini M., Jafarnejad S., Vahedpoor Z., Asemi Z. (2019). The effects of curcumin-containing supplements on biomarkers of inflammation and oxidative stress: A systematic review and meta-analysis of randomized controlled trials. Phyther. Res..

[25] Rahban M., Habibi-Rezaei M., Mazaheri M., Saso L., Moosavi-Movahedi A.A. (2020). Anti-viral potential and modulation of nrf2 by curcumin: Pharmacological implications. Antioxidants.

[26] Basak S., Srinivas V., Mallepogu A., Duttaroy A.K. (2020). Curcumin stimulates angiogenesis through VEGF and expression of HLA-G in first-trimester human placental trophoblasts. Cell Biol. Int..

[27] Qi L., Jiang J., Zhang J., Zhang L., Wang T. (2020). Curcumin protects human trophoblast HTR8/SVneo cells from H2O2-induced oxidative stress by activating nrf2 signaling pathway. Antioxidants.

[28] Fadinie W., Lelo A., Wijaya D.W., Lumbanraja S.N. (2019). Curcumin’s effect on COX-2 and IL-10 serum in preeclampsia’s patient undergo sectio caesarea with spinal anesthesia. Open Access Maced. J. Med. Sci..

[29] Sun J., Zhong W., Gu Y., Groome L.J., Wang Y. (2014). 1,25(OH)2D3 suppresses COX-2 up-regulation and thromboxane production in placental trophoblast cells in response to hypoxic stimulation. Placenta.

[30] Gong P., Liu M., Hong G., Li Y., Xue P., Zheng M., Wu M., Shen L., Yang M., Diao Z. (2016). Curcumin improves LPS-induced preeclampsia-like phenotype in rat by inhibiting the TLR4 signaling pathway. Placenta.

[31] Zhou J., Miao H., Li X., Hu Y., Sun H., Hou Y. (2017). Curcumin inhibits placental inflammation to ameliorate LPS-induced adverse pregnancy outcomes in mice via upregulation of phosphorylated Akt. Inflamm. Res..

[32] Qi L., Jiang J., Zhang J., Zhang L., Wang T. (2020). Maternal curcumin supplementation ameliorates placental function and fetal growth in mice with intrauterine growth retardation. Biol. Reprod..

[33] Shi D.D., Guo J.J., Zhou L., Wang N. (2018). Epigallocatechin gallate enhances treatment efficacy of oral nifedipine against pregnancy-induced severe pre-eclampsia: A double-blind, randomized and placebo-controlled clinical study. J. Clin. Pharm. Ther..

[34] Hobson S.R., Gurusinghe S., Lim R., Alers N.O., Miller S.L., Kingdom J.C., Wallace E.M. (2018). Melatonin improves endothelial function in vitro and prolongs pregnancy in women with early-onset preeclampsia. J. Pineal Res..

[35] Ding J., Kang Y., Fan Y., Chen Q. (2017). Efficacy of resveratrol to supplement oral nifedipine treatment in pregnancy-induced preeclampsia. Endocr. Connect..

[36] Caldeira-Dias M., Montenegro M.F., Bettiol H., Barbieri M.A., Cardoso V.C., Cavalli R.C., Sandrim V.C. (2019). Resveratrol improves endothelial cell markers impaired by plasma incubation from women who subsequently develop preeclampsia. Hypertens. Res..

[37] Caldeira-Dias M., Viana-Mattioli S., de Souza Rangel Machado J., Carlström M., de Carvalho Cavalli R., Sandrim V.C. (2021). Resveratrol and grape juice: Effects on redox status and nitric oxide production of endothelial cells in in vitro preeclampsia model. Pregnancy Hypertens..

[38] Miller S.L., Yawno T., Alers N.O., Castillo-Melendez M., Supramaniam V.G., Vanzyl N., Sabaretnam T., Loose J.M., Drummond G.R., Walker D.W. (2014). Antenatal antioxidant treatment with melatonin to decrease newborn neurodevelopmental deficits and brain injury caused by fetal growth restriction. J. Pineal Res..

[39] De la Torre R., de Sola S., Hernandez G., Farré M., Pujol J., Rodriguez J., Espadaler J.M., Langohr K., Cuenca-Royo A., Principe A. (2016). Safety and efficacy of cognitive training plus epigallocatechin-3-gallate in young adults with Down’s syndrome (TESDAD): A double-blind, randomised, placebo-controlled, phase 2 trial. Lancet Neurol..

[40] Quezada-Fernández P., Trujillo-Quiros J., Pascoe-González S., Trujillo-Rangel W.A., Cardona-Müller D., Ramos-Becerra C.G., Barocio-Pantoja M., Rodríguez-de la Cerda M., Nérida Sánchez-Rodríguez E., Cardona-Muñóz E.G. (2019). Effect of green tea extract on arterial stiffness, lipid profile and sRAGE in patients with type 2 diabetes mellitus: A randomised, double-blind, placebo-controlled trial. Int. J. Food Sci. Nutr..

[41] Fernandes R.C., Araújo V.A., Giglio B.M., Mota J.F., Teixeira K.I.S.S., Monteiro P.A., Lira F.S., Pimentel G.D. (2018). Acute epigallocatechin 3 gallate (EGCG) supplementation delays gastric emptying in healthy women: A randomized, double-blind, placebo-controlled crossover study. Nutrients.

[42] Chen I.J., Liu C.Y., Chiu J.P., Hsu C.H. (2016). Therapeutic effect of high-dose green tea extract on weight reduction: A randomized, double-blind, placebo-controlled clinical trial. Clin. Nutr..

[43] Fernández V.A., Toledano L.A., Lozano N.P., Tapia E.N., Roig M.D.G., Fornell R.D.L.T., Algar Ó.G. (2020). Bioavailability of epigallocatechin gallate administered with different nutritional strategies in healthy volunteers. Antioxidants.

[44] Zhang H., Su S., Yu X., Li Y. (2017). Dietary epigallocatechin 3-gallate supplement improves maternal and neonatal treatment outcome of gestational diabetes mellitus: A double-blind randomised controlled trial. J. Hum. Nutr. Diet..

[45] Zhong M., Peng J., Xiang L., Yang X., Wang X., Zhu Y. (2020). Epigallocatechin gallate (EGCG) improves anti-angiogenic state, cell viability, and hypoxia-induced endothelial dysfunction by downregulating high mobility group Box 1 (HMGB1) in preeclampsia. Med. Sci. Monit..

[46] Sava R.I., March K.L., Pepine C.J. (2018). Hypertension in pregnancy: Taking cues from pathophysiology for clinical practice. Clin. Cardiol..

[47] Bellos I., Karageorgiou V., Kapnias D., Karamanli K.E., Siristatidis C. (2018). The role of interleukins in preeclampsia: A comprehensive review. Am. J. Reprod. Immunol..

[48] Almeida-Toledano L., Andreu-Fernández V., Aras-López R., García-Algar Ó., Martínez L., Gómez-Roig M.D. (2021). Epigallocatechin gallate ameliorates the effects of prenatal alcohol exposure in a fetal alcohol spectrum disorder-like mouse model. Int. J. Mol. Sci..

[49] Chen L.H., Wu M., Hu X.H., Wang Y.F. (2020). Effect of epigallocatechin-3-gallate on liver lipid metabolism in rats with intrauterine growth restriction and related mechanism. Chin. J. Contemp. Pediatr..

[50] Kumar A., Gupta A., Malhotra V.K., Agarwal P.S., Thirupuram S., Gaind B. (1989). Cord blood lipid levels in low birth weight newborns. Indian Pediatr..

[51] Molina S.M., Casanueva E.V., Cid C.X., Ferrada N.M.C., Pérez V.R., Dios T.G., Reyes R.M., Venegas B.H., Cid S.L. (2000). Lipid profile in newborns with intrauterine growth retardation. Rev. Med. Chile.

[52] Boocock D.J., Faust G.E.S., Patel K.R., Schinas A.M., Brown V.A., Ducharme M.P., Booth T.D., Crowell J.A., Perloff M., Gescher A.J. (2007). Phase I dose escalation pharmacokinetic study in healthy volunteers of resveratrol, a potential cancer chemopreventive agent. Cancer Epidemiol. Biomark. Prev..

[53] Roberts V.H.J., Pound L.D., Thorn S.R., Gillingham M.B., Thornburg K.L., Friedman J.E., Frias A.E., Grove K.L. (2014). Beneficial and cautionary outcomes of resveratrol supplementation in pregnant nonhuman primates. FASEB J..

[54] Bourque S.L., Dolinsky V.W., Dyck J.R.B., Davidge S.T. (2012). Maternal resveratrol treatment during pregnancy improves adverse fetal outcomes in a rat model of severe hypoxia. Placenta.

[55] Levine A.B., Punihaole D., Levine T.B. (2012). Characterization of the role of nitric oxide and its clinical applications. Cardiology.

[56] George E.M., Granger J.P. (2013). Heme oxygenase in pregnancy and preeclampsia. Curr. Opin. Nephrol. Hypertens..

[57] Poudel R., Stanley J.L., Rueda-Clausen C.F., Andersson I.J., Sibley C.P., Davidge S.T., Baker P.N. (2013). Effects of Resveratrol in Pregnancy Using Murine Models with Reduced Blood Supply to the Uterus. PLoS ONE.

[58] Zou Y., Zuo Q., Huang S., Yu X., Jiang Z., Zou S., Fan M., Sun L. (2014). Resveratrol inhibits trophoblast apoptosis through oxidative stress in preeclampsia-model rats. Molecules.

[59] Zou Y., Li S., Wu D., Xu Y., Wang S., Jiang Y., Liu F., Jiang Z., Qu H., Yu X. (2019). Resveratrol promotes trophoblast invasion in pre-eclampsia by inducing epithelial-mesenchymal transition. J. Cell. Mol. Med..

[60] Darby J.R.T., Saini B.S., Soo J.Y., Lock M.C., Holman S.L., Bradshaw E.L., McInnes S.J.P., Voelcker N.H., Macgowan C.K., Seed M. (2019). Subcutaneous maternal resveratrol treatment increases uterine artery blood flow in the pregnant ewe and increases fetal but not cardiac growth. J. Physiol..

[61] Rodrigues Helmo F., Etchebehere R.M., Bernardes N., Meirelles M.F., Galvão Petrini C., Penna Rocha L., Gonçalves dos Reis Monteiro M.L., Souza de Oliveira Guimarães C., de Paula Antunes Teixeira V., dos Reis M.A. (2018). Melatonin treatment in fetal and neonatal diseases. Pathol. Res. Pract..

[62] Okatani Y., Okamoto K., Hayashi K., Wakatsuki A., Tamura S., Sagara Y. (1998). Maternal-fetal transfer of melatonin in pregnant women near term. J. Pineal Res..

[63] Dou Y., Lin B., Cheng H., Wang C., Zhao M., Zhang J., Wu J. (2019). The reduction of melatonin levels is associated with the development of preeclampsia: A meta-analysis. Hypertens. Pregnancy.

[64] Berbets A.M., Davydenko I.S., Barbe A.M., Konkov D.H., Albota O.M., Yuzko O.M. (2021). Melatonin 1A and 1B Receptors’ Expression Decreases in the Placenta of Women with Fetal Growth Restriction. Reprod. Sci..

[65] Berbets A.M., Barbe A.M., Andriiets O.A., Andriiets A.V., Yuzko O.M. (2020). Melatonin Levels Decrease in the Umbilical Cord in Case of Intrauterine Growth Restriction. J. Med. Life.

[66] Berbets A., Koval H., Barbe A., Albota O., Yuzko O. (2021). Melatonin decreases and cytokines increase in women with placental insufficiency. J. Matern. Neonatal Med..

[67] Hobson S.R., Lim R., Gardiner E.E., Alers N.O., Wallace E.M. (2013). Phase I pilot clinical trial of antenatal maternally administered melatonin to decrease the level of oxidative stress in human pregnancies affected by pre-eclampsia (PAMPR): Study protocol. BMJ Open.

[68] El-Malkey N.F., Aref M., Emam H., Khalil S.S. (2021). Impact of Melatonin on Full-Term Fetal Brain Development and Transforming Growth Factor-β Level in a Rat Model of Preeclampsia. Reprod. Sci..

[69] Zuo J., Jiang Z. (2020). Melatonin attenuates hypertension and oxidative stress in a rat model of L-NAME-induced gestational hypertension. Vasc. Med..

[70] Uzun M., Gencer M., Turkon H., Oztopuz R.O., Demir U., Ovali M.A. (2017). Effects of Melatonin on Blood Pressure, Oxidative Stress and Placental Expressions of TNFα, IL-6, VEGF and sFlt-1 in RUPP Rat Model of Preeclampsia. Arch. Med. Res..

[71] Doğanlar O., Doğanlar Z.B., Ovali M.A., Güçlü O., Demir U., Doğan A., Uzun M. (2020). Melatonin regulates oxidative stress and apoptosis in fetal hearts of pinealectomised RUPP rats. Hypertens. Pregnancy.

[72] Tain Y.L., Huang L.T., Hsu C.N., Lee C. (2014). Te Melatonin therapy prevents programmed hypertension and nitric oxide deficiency in offspring exposed to maternal caloric restriction. Oxid. Med. Cell. Longev..

[73] Tain Y.L., Leu S., Wu K.L.H., Lee W.C., Chan J.Y.H. (2014). Melatonin prevents maternal fructose intake-induced programmed hypertension in the offspring: Roles of nitric oxide and arachidonic acid metabolites. J. Pineal Res..

[74] Zhu H.L., Shi X.T., Xu X.F., Zhou G.X., Xiong Y.W., Yi S.J., Liu W.B., Dai L.M., Cao X.L., Xu D.X. (2021). Melatonin protects against environmental stress-induced fetal growth restriction via suppressing ROS-mediated GCN2/ATF4/BNIP3-dependent mitophagy in placental trophoblasts. Redox Biol..

[75] Tare M., Parkington H.C., Wallace E.M., Sutherland A.E., Lim R., Yawno T., Coleman H.A., Jenkin G., Miller S.L. (2014). Maternal melatonin administration mitigates coronary stiffness and endothelial dysfunction, and improves heart resilience to insult in growth restricted lambs. J. Physiol..

[76] Sales F., Peralta O.A., Narbona E., Mccoard S., González-Bulnes A., Parraguez V.H. (2019). Rapid Communication: Maternal melatonin implants improve fetal oxygen supply and body weight at term in sheep pregnancies. J. Anim. Sci..

[77] Lemley C.O., Meyer A.M., Camacho L.E., Neville T.L., Newman D.J., Caton J.S., Vonnahme K.A. (2012). Melatonin supplementation alters uteroplacental hemodynamics and fetal development in an ovine model of intrauterine growth restriction. Am. J. Physiol.-Regul. Integr. Comp. Physiol..

[78] Lemley C.O., Camacho L.E., Meyer A.M., Kapphahn M., Caton J.S., Vonnahme K.A. (2013). Dietary melatonin supplementation alters uteroplacental amino acid flux during intrauterine growth restriction in ewes. Animal.

[79] Aynaoglu Yildiz G., Yildiz D., Yapca O.E., Suleyman B., Arslan Y.K., Kurt N., Suleyman H. (2021). Effect of diazepam, sertraline and melatonin on the stress-induced reproductive disorders and intrauterine growth restriction in female rats. J. Matern. Neonatal Med..

[80] Castillo-Melendez M., Yawno T., Sutherland A., Jenkin G., Wallace E.M., Miller S.L. (2017). Effects of Antenatal Melatonin Treatment on the Cerebral Vasculature in an Ovine Model of Fetal Growth Restriction. Dev. Neurosci..

[81] Polglase G.R., Barbuto J., Allison B.J., Yawno T., Sutherland A.E., Malhotra A., Schulze K.E., Wallace E.M., Jenkin G., Ricardo S.D. (2017). Effects of antenatal melatonin therapy on lung structure in growth-restricted newborn lambs. J. Appl. Physiol..

[82] González-Candia A., Veliz M., Araya C., Quezada S., Ebensperger G., Serón-Ferré M., Reyes R.V., Llanos A.J., Herrera E.A. (2016). Potential adverse effects of antenatal melatonin as a treatment for intrauterine growth restriction: Findings in pregnant sheep. Am. J. Obstet. Gynecol..

[83] Palmer K.R., Mockler J.C., Davies-Tuck M.L., Miller S.L., Goergen S.K., Fahey M.C., Anderson P.J., Groom K.M., Wallace E.M. (2019). Protect-me: A parallel-group, triple blinded, placebo-controlled randomised clinical trial protocol assessing antenatal maternal melatonin supplementation for fetal neuroprotection in early-onset fetal growth restriction. BMJ Open.

[84] Harrison M.S., Goldenberg R.L. (2016). Global burden of prematurity. Semin. Fetal Neonatal Med..

[85] Martin C.L., Sotres-Alvarez D., Siega-Riz A.M. (2015). Maternal dietary patterns during the second trimester are associated with preterm birth. J. Nutr..

[86] Ghomian N., Hafizi L.T.Z. (2013). The role of vitamin C in prevention of preterm premature rupture of membranes. Iran. Red Crescent Med. J..

[87] Gupta S., Gaikwad H.S., Nath B., Batra A. (2020). Can vitamin C and interleukin 6 levels predict preterm premature rupture of membranes: Evaluating possibilities in North Indian population. Obstet. Gynecol. Sci..

[88] Sharma R., Mehta S. (2014). Ascorbic Acid Concentration and Preterm Premature Rupture of Membranes. J. Obstet. Gynecol. India.

[89] Harville E.W., Lewis C.E., Catov J.M., Jacobs D.R., Gross M.D., Gunderson E.P. (2020). A longitudinal study of pre-pregnancy antioxidant levels and subsequent perinatal outcomes in black and white women: The CARDIA study. PLoS ONE.

[90] Bártfai L., Bártfai Z., Nedeczky I., Puho E.H., Bánhidy F., Czeizel A.E. (2012). Rate of preterm birth in pregnant women with vitamin e treatment: A population-based study. J. Matern. Neonatal Med..

[91] Carmichael S., Yang W., Shaw G. (2013). Maternal dietary nutrient intake and risk of preterm delivery. Am. J. Perinatol..

[92] Gungorduk K., Asicioglu O., Gungorduk O.C., Yildirim G., Besimoǧlu B., Ark C. (2014). Does vitamin C and vitamin E supplementation prolong the latency period before delivery following the preterm premature rupture of membranes? A randomized controlled study. Am. J. Perinatol..

[93] Hassanzadeh A., Paknahad Z., Khoigani M. (2016). The relationship between macro- and micro-nutrients intake and risk of preterm premature rupture of membranes in pregnant women of Isfahan. Adv. Biomed. Res..

[94] Hauth J.C., Clifton R.G., Roberts J.M., Spong C.Y., Myatt L., Leveno K.J., Pearson G.D., Varner M.W., Thorp J.M., Mercer B.M. (2010). Vitamin C and E supplementation to prevent spontaneous preterm birth: A randomized controlled trial. Obstet. Gynecol..

[95] Ilhan N., Aygun B.K., Gungor H. (2017). The relationship between the latency period, infection markers, and oxidant and antioxidant states in women with preterm premature rupture of membranes. Ir. J. Med. Sci..

[96] Koenig M.D., McFarlin B.L., Steffen A.D., Tussing-Humphreys L., Giurgescu C., Engeland C.G., Kominiarek M.A., Ciezczak-Karpiel C., O’Brien W.D., White-Traut R. (2017). Decreased Nutrient Intake Is Associated With Premature Cervical Remodeling. JOGNN-J. Obstet. Gynecol. Neonatal Nurs..

[97] Zhang Y., Zhou H., Perkins A., Wang Y., Sun J. (2017). Maternal dietary nutrient intake and its association with preterm birth: A case-control study in Beijing, China. Nutrients.

[98] Charkamyani F., Khedmat L., Hosseinkhani A. (2021). Decreasing the main maternal and fetal complications in women undergoing in vitro fertilization (IVF) trained by nutrition and healthy eating practices during pregnancy. J. Matern. Neonatal Med..

[99] Nga H.T., Quyen P.N., Chaffee B.W., Diep Anh N.T., Ngu T., King J.C. (2020). Effect of a nutrient-rich, food-based supplement given to rural Vietnamese mothers prior to and/or during pregnancy on birth outcomes: A randomized controlled trial. PLoS ONE.

[100] Nossier S.A., Naeim N.E., El-Sayed N.A., Abu Zeid A.A. (2015). The effect of zinc supplementation on pregnancy outcomes: A double-blind, randomised controlled trial, Egypt. Br. J. Nutr..

[101] Zahiri Sorouri Z., Sadeghi H., Pourmarzi D. (2016). The effect of zinc supplementation on pregnancy outcome: A randomized controlled trial. J. Matern. Neonatal Med..

[102] Costa M.M., Belo S., Souteiro P., Neves J.S., Magalhães D., Silva R.B., Oliveira S.C., Freitas P., Varela A., Queirós J. (2018). Pregnancy after bariatric surgery: Maternal and fetal outcomes of 39 pregnancies and a literature review. J. Obstet. Gynaecol. Res..

[103] Kucukaydin Z., Kurdoglu M., Kurdoglu Z., Demir H., Yoruk I.H. (2018). Selected maternal, fetal and placental trace element and heavy metal and maternal vitamin levels in preterm deliveries with or without preterm premature rupture of membranes. J. Obstet. Gynaecol. Res..

[104] Shen P.J., Gong B., Xu F.Y., Luo Y. (2015). Four trace elements in pregnant women and their relationships with adverse pregnancy outcomes. Eur. Rev. Med. Pharmacol. Sci..

[105] Chen L.W., Fitzgerald R., Murrin C.M., Mehegan J., Kelleher C.C., Phillips C.M. (2018). Associations of maternal caffeine intake with birth outcomes: Results from the Lifeways Cross Generation Cohort Study. Am. J. Clin. Nutr..

[106] Huang L., Lerro C., Yang T., Li J., Qiu J., Qiu W., He X., Cui H., Lv L., Xu R. (2016). Maternal tea consumption and the risk of preterm delivery in urban China: A birth cohort study. BMC Public Health.

[107] Lu J.H., He J.R., Shen S.Y., Wei X.L., Chen N.N., Yuan M.Y., Qiu L., Li W.D., Chen Q.Z., Hu C.Y. (2017). Does tea consumption during early pregnancy have an adverse effect on birth outcomes?. Birth.

[108] Okubo H., Miyake Y., Tanaka K., Sasaki S., Hirota Y. (2015). Maternal total caffeine intake, mainly from Japanese and Chinese tea, during pregnancy was associated with risk of preterm birth: The Osaka Maternal and Child Health Study. Nutr. Res..

[109] Moussally K., Bérard A. (2010). Exposure to herbal products during pregnancy and the risk of preterm birth. Eur. J. Obstet. Gynecol. Reprod. Biol..

[110] Sengpiel V., Elind E., Bacelis J., Nilsson S., Grove J., Myhre R., Haugen M., Meltzer H.M., Alexander J., Jacobsson B. (2013). Maternal caffeine intake during pregnancy is associated with birth weight but not with gestational length: Results from a large prospective observational cohort study. BMC Med..

[111] Sindiani A.M., Khader Y., Amarin Z. (2020). The association between coffee and tea consumption during pregnancy and preterm delivery: Case–control study. J. Multidiscip. Healthc..

[112] Biran V., Decobert F., Bednarek N., Boizeau P., Benoist J.F., Claustrat B., Barré J., Colella M., Frérot A., Garnotel R. (2019). Melatonin levels in preterm and term infants and their mothers. Int. J. Mol. Sci..

[113] Domínguez Rubio A.P., Sordelli M.S., Salazar A.I., Aisemberg J., Bariani M.V., Cella M., Rosenstein R.E., Franchi A.M. (2014). Melatonin prevents experimental preterm labor and increases offspring survival. J. Pineal Res..

[114] Lee J.Y., Song H., Dash O., Park M., Shin N.E., McLane M.W., Lei J., Hwang J.Y., Burd I. (2019). Administration of melatonin for prevention of preterm birth and fetal brain injury associated with premature birth in a mouse model. Am. J. Reprod. Immunol..

[115] Ramiro-Cortijo D., de la Calle M., Rodríguez-Rodríguez P., López de Pablo Á.L., López-Giménez M.R., Aguilera Y., Martín-Cabrejas M.A., González M.D.C., Arribas S.M. (2020). Maternal antioxidant status in early pregnancy and development of fetal complications in twin pregnancies: A pilot study. Antioxidants.

[116] Specht I.O., Hammer P.E.C., Flachs E.M., Begtrup L.M., Larsen A.D., Hougaard K.S., Hansen J., Hansen Å.M., Kolstad H.A., Rugulies R. (2019). Night work during pregnancy and preterm birth—A large register-based cohort study. PLoS ONE.

[117] Robins J.C., Marsit C.J., Padbury J.F., Sharma S.S. (2011). Endocrine disruptors, environmental oxygen, epigenetics and pregnancy. Front. Biosci.-Elit. Ed..

[118] Burton G.J., Cindrova-Davies T., Yung H.W., Jauniaux E. (2021). Hypoxia and reproductive health: Oxygen and development of the human placenta. Reproduction.

[119] Lorenzetti S., Plösch T., Teller I.C. (2021). Antioxidative molecules in human milk and environmental contaminants. Antioxidants.

[120] Hoppu U., Rinne M., Salo-Väänänen P., Lampi A.M., Piironen V., Isolauri E. (2005). Vitamin C in breast milk may reduce the risk of atopy in the infant. Eur. J. Clin. Nutr..

[121] Zarban A., Toroghi M.M., Asli M., Jafari M., Vejdan M., Sharifzadeh G. (2015). Effect of vitamin C and e supplementation on total antioxidant content of human breastmilk and infant Urine. Breastfeed. Med..

[122] Daneel-Otterbech S., Davidsson L., Hurrell R. (2005). Ascorbic acid supplementation and regular consumption of fresh orange juice increase the ascorbic acid content of human milk: Studies in European and African lactating women. Am. J. Clin. Nutr..

[123] Friel J.K., Diehl-Jones W.L., Suh M., Tsopmo A., Shirwadkar V.P. (2007). Impact of iron and vitamin C-containing supplements on preterm human milk: In vitro. Free Radic. Biol. Med..

[124] Da Silva A.L.C., Da Silva Ribeiro K.D., De Melo L.R.M., Bezerra D.F., De Queiroz J.L.C., Lima M.S.R., Pires J.F., Bezerra D.S., Osório M.M., Dimenstein R. (2017). Vitamin E in human milk and its relation to the nutritional requirement of the term newborn. Rev. Paul. Pediatr..

[125] De Melo L.R.M., Clemente H.A., Bezerra D.F., Dantas R.C.S., Ramalho H.M.M., Dimenstein R. (2017). Effect of maternal supplementation with vitamin E on the concentration of α-tocopherol in colostrum. J. Pediatr..

[126] Pires Medeiros J.F., Ribeiro K.D.D.S., Lima M.S.R., Das Neves R.A.M., Lima A.C.P., Dantas R.C.S., Da Silva A.B., Dimenstein R. (2016). α-Tocopherol in breast milk of women with preterm delivery after a single postpartum oral dose of vitamin e. Br. J. Nutr..

[127] Keikha M., Shayan-Moghadam R., Bahreynian M., Kelishadi R. (2021). Nutritional supplements and mother’s milk composition: A systematic review of interventional studies. Int. Breastfeed. J..

[128] Darlow B.A., Austin N. (2003). Selenium supplementation to prevent short-term morbidity in preterm neonates. Cochrane Database Syst. Rev..

[129] Strambi M., Longini M., Vezzosi P., Berni S., Buoni S. (2004). Selenium status, birth weight, and breast-feeding: Pattern in the first month. Biol. Trace Elem. Res..

[130] Shaaban S.Y., El-Hodhod M.A.A., Nassar M.F., Hegazy A.E.T., El-Arab S.E., Shaheen F.M. (2005). Zinc status of lactating Egyptian mothers and their infants: Effect of maternal zinc supplementation. Nutr. Res..

[131] Loui A., Raab A., Wagner M., Weigel H., Grüters-Kieslich A., Brätter P., Obladen M. (2004). Nutrition of very low birth weight infants fed human milk with or without supplemental trace elements: A randomized controlled trial. J. Pediatr. Gastroenterol. Nutr..

[132] Sánchez-Barceló E.J., Mediavilla M.D., Reiter R.J. (2011). Clinical Uses of Melatonin in Pediatrics. Int. J. Pediatr..

[133] Qin Y., Shi W., Zhuang J., Liu Y., Tang L., Bu J., Sun J., Bei F. (2019). Variations in melatonin levels in preterm and term human breast milk during the first month after delivery. Sci. Rep..

[134] Honorio-França A.C., Hara C.C.P., Ormonde J.V.S., Nunes G.T., França E.L. (2013). Human colostrum melatonin exhibits a day-night variation and modulates the activity of colostral phagocytes. J. Appl. Biomed..

[135] Clemente H.A., Ramalho H.M.M., Lima M.S.R., Grilo E.C., Dimenstein R. (2015). Maternal supplementation with natural or synthetic vitamin E and its levels in human colostrum. J. Pediatr. Gastroenterol. Nutr..

[136] Simon-szabo Z., Fogarasi E., Nemes-Nagy E., Denes L., Croitoru M., Szabo B. (2021). Oxidative stress and peripartum outcomes (Review). Exp. Ther. Med..

[137] Singh S., Aggarwal B.B. (1995). Activation of transcription factor NF-κB is suppressed by curcumin (diferulolylmethane). J. Biol. Chem..

[138] Walsh S.W. (1985). Preeclampsia: An imbalance in placental prostacyclin and thromboxane Production. Am. J. Obstet. Gynecol..

[139] Cas M.D., Ghidoni R. (2019). Dietary curcumin: Correlation between bioavailability and health potential. Nutrients.

[140] Guillier C., Carrière D., Pansiot J., Maroni A., Billion E., Ringot M., Benoist J.F., Jacques S., Matrot B., Jarreau P.H. (2021). Nebulized curcumin protects neonatal lungs from antenatal insult in rats. Am. J. Physiol.-Lung Cell. Mol. Physiol..

[141] Der Hsuuw Y., Chang C.K., Chan W.H., Yu J.S. (2005). Curcumin prevents methylglyoxal-induced oxidative stress and apoptosis in mouse embryonic stem cells and blastocysts. J. Cell. Physiol..

[142] Ganiger S., Malleshappa H.N., Krishnappa H., Rajashekhar G., Ramakrishna Rao V., Sullivan F. (2007). A two generation reproductive toxicity study with curcumin, turmeric yellow, in Wistar rats. Food Chem. Toxicol..

[143] Malvasi A., Kosmas I., Mynbaev O.A., Sparic R., Gustapane S., Guido M., Tinelli A. (2017). Can trans resveratrol plus d-chiro-inositol and myo-inositol improve maternal metabolic profile in overweight pregnant patients?. Clin. Ter..

[144] Viana-Mattioli S., Cinegaglia N., Bertozzi-Matheus M., Bueno-Pereira T.O., Caldeira-Dias M., Cavalli R.C., Sandrim V.C. (2020). SIRT1-dependent effects of resveratrol and grape juice in an in vitro model of preeclampsia. Biomed. Pharmacother..

[145] Dai B., Liu T., Zhang B., Zhang X., Wang Z. (2013). The polymorphism for endothelial nitric oxide synthase gene, the level of nitric oxide and the risk for pre-eclampsia: A meta-analysis. Gene.

[146] Cui L., Xu F., Wang S., Jiang Z., Liu L., Ding Y., Sun X., Du M. (2021). Melatonin-MT1 signal is essential for endometrial decidualization. Reproduction.

[147] Von Dadelszen P., Ornstein M.P., Bull S.B., Logan A.G., Koren G., Magee L.A. (2000). Fail in mean arterial pressure and fetal growth restriction in pregnancy hypertension: A meta-analysis. Lancet.

[148] Ottosen L.D.M., Hindkjær J., Husth M., Petersen D.E., Kirk J., Ingerslev H.J. (2006). Observations on intrauterine oxygen tension measured by fibre-optic microsensors. Reprod. Biomed. Online.

[149] Bravi F., Wiens F., Decarli A., Dal Pont A., Agostoni C., Ferraroni M. (2016). Impact of maternal nutrition on breast-milk composition: A systematic review. Am. J. Clin. Nutr..

[150] Shkayeva M., Gregory P., Pickering M., Hein D., Hu J., Rodriguez A. (2015). Green Tea Product Epigallocatechin Gallate (EGCG) Content and Label Information: A Descriptive Analysis. J. Nutr. Ther..

[151] Rossi D., Guerrini A., Bruni R., Brognara E., Borgatti M., Gambari R., Maietti S., Sacchetti G. (2012). Trans-resveratrol in nutraceuticals: Issues in retail quality and effectiveness. Molecules.

[152] Moraloglu O., Engin-Ustun Y., Tonguç E., Var T., Tapisiz Ö.L., Ergün H., Guvenc T., Gacar A. (2012). The effect of resveratrol on blood pressure in a rat model of preeclampsia. J. Matern. Neonatal Med..

[153] Renshall L.J., Morgan H.L., Moens H., Cansfield D., Finn-Sell S.L., Tropea T., Cottrell E.C., Greenwood S., Sibley C.P., Wareing M. (2018). Melatonin increases fetal weight in wild-type mice but not in mouse models of fetal growth restriction. Front. Physiol..

[154] Moallem S.A., Afshar M., Etemad L., Razavi B.M., Hosseinzadeh H. (2016). Evaluation of teratogenic effects of crocin and safranal, active ingredients of saffron, in mice. Toxicol. Ind. Health.

[155] Strakovsky R.S., Pan Y.X. (2012). In utero oxidative stress epigenetically programs antioxidant defense capacity and adulthood diseases. Antioxid. Redox Signal..

