# Supplementary material for: Effects of Antioxidant Intake on Fetal Development and Maternal/Neonatal Health during Pregnancy
Source: Antioxidants (Basel). 2022 Mar 28;11(4):648. doi: 10.3390/antiox11040648 (PMC9028185; doi:10.3390/antiox11040648)
Supplement: Supplementary file 1 [file antioxidants-11-00648-s001.zip › antioxidants-1636668-supplementary.pdf]

**Supplemental Table S1.** Grading of Recommendations Assessment, Development, and Evaluation (GRADE) of selected studies.

| Quality assessment | | | | | | Nº of patients | | Effect | | Quality | Importance |
|---|---|---|---|---|---|---|---|---|---|---|---|
| **Nº of studies** | **Design** | **Risk of bias** | **Inconsistency** | **Indirectness** | **Imprecision** | **Cases** | **Control** | **Relative (95%CI)** | **Absolute** | | |
| **Fadinie (2019) [28]. Curcumin and PE (90 min of follow-up )** | | | | | | | | | | | |
| **1** | RCCT | Serious risk of bias (no information about randomization) | No Serious inconsistency | Serious indirectness ( no population data at baseline) | Serious imprecision (very wide range of results) | 23 | 24 | No significant effects on IL-10 or COX-2 serum levels | - | Low ++/++++ | Less important |
| **Shi (2018) [33]; Ding (2017) [35]. EGCG/Resveratrol and Nifedipine on PE (follow-up until normalization of BP )** | | | | | | | | | | | |
| **2** | RCCT | No Serious risk of bias | No Serious inconsistency | No serious indirectness | No serious imprecision | 322 | 331 | Time to control BP: Mean difference of 14.1 min (9.7-18.5; 95% CI) and 15.5 min (4.7-10.9; 95% CI) for EGCG and RESV, respectively<br><br>Time until new HC: Mean difference of 3.1 | - | High ++++/++++ | Critical |

| | | | | | | | | | | | |
|---|---|---|---|---|---|---|---|---|---|---|---|
| | | | | | | | | h (2.3-3.9; 95% CI) and 2.5 h (0.3-2.4; 95% CI) for EGCG and RESV, respectively | | | |
| **Hobson (2018)** [34]. **Melatonin an PE (follow-up from recruitment until delivery)** | | | | | | | | | | | |
| **1** | Phase I single arm clinical trial | Serious risk of bias (unblinding, no randomization) | No Serious inconsistency | Serious indirectness (use of historical controls) | No serious imprecision | 20 | 48 | No significant effects on mean arterial BP Interval from diagnosis to delivery: mean 6 [± 2.3 SEM] days; P<0.05) vs HCC | - | Low ++/++++ | Important |
| **Caldeira-Dias (2019)** [36]. **Resveratrol and PE (No follow-up)** | | | | | | | | | | | |
| **1** | Observational | Serious risk of bias (unblinding, lack of information on the choice of patients) | No Serious inconsistency | Serious indirectness | Serious imprecision (low population) | 6 | 6 | Improvement of HO-1 and NO markers and GSH levels in HUVECs treated with RESV | - | Very Low +/++++ | Less Important |
| **Caldeira-Dias (2021)** [37]. **Grape juice and PE (follow-up of 1 hour)** | | | | | | | | | | | |
| **1** | Phase I single arm open-label | Serious risk of bias (unblinding, no randomization) | No serious inconsistency | Serious indirectness ( no population data at baseline) | Serious imprecision (low population) | 4 | - | 17% ± 4, 50%± 2 and 69%± 7 decrease in HO-1, GSH and ARE activity levels, respectively, | - | Very Low +/++++ | Important |

| | | | | | | | | | | | |
|---|---|---|---|---|---|---|---|---|---|---|---|
| | | | | | | | | after treatment (p<0.05) in HUVECs | | | |
| **Miller (2014)** [38]**. Melatotin and fetal growth restriction (follow-up from recruitment until delivery)** | | | | | | | | | | | |
| **1** | Phase I single arm open-label | Serious risk of bias (unblinding, no randomization) | No serious inconsistency | Serious indirectness (use of historical controls) | Serious imprecision (low population) | 6 | 6 | Significant lower MDA concentration (2.4 ± 0.6 nmol/mg tissue) versus control (4.6 ± 0.7 nmol/mg tissue; P = 0.04) | - | Low ++/++++ | Important |
| **Ghomian (2013)** [86]**, Gupta (2020)** [87]**. Vitamin C use and reduction in PPROM** | | | | | | | | | | | |
| **1** | RCCT | Serious risk of bias (blinding) | No serious inconsistency | No serious indirectness | No serious imprecision | 85 | 85 | RR 0.70 | OR 0.31 (vit C) OR 0.44 (controls) | Moderate +++/++++ | Critical |
| **1** | Case control study | Undetected | No serious inconsistency | No serious indirectness | No serious imprecision | 20 | 20 | RR 0.18 | OR 0.12 (vit C) OR 0.66 (controls) | Low ++/++++ | Critical |
| **Sharma (2014)** [88]**, Ilhan (2107)** [95]**, Ilhan (2015)** [15]**, Hassanzadeh (2016)** [93]**. Plasmatic ascorbic acid levels in PPROM** | | | | | | | | | | | |

| | | | | | | | | | | | |
|---|---|---|---|---|---|---|---|---|---|---|---|
| **2** | Case control study | Undetected | No serious inconsistency | No serious indirectness | No serious imprecision | 95 | 61 | - | 0.41-7.39 mg/L (PPROM) 0.84-13.83 mg/L (controls) | Low ++/++++ | Important but not critical |
| **1** | Cohort study | Undetected | No serious inconsistency | No serious indirectness | No serious imprecision | 620 | | - | OR 1.001 (vit C) | Low ++/++++ | Important but not critical |
| **1** | Cross-sectional study | Undetected | No serious inconsistency | No serious indirectness | No serious imprecision | 38 | 34 | - | 7.29±2.19 mg/L (PPROM) 13.85±3.07 mg/L (controls) | Low ++/++++ | Important but not critical |
| **Martin (2015)** [85]**. Vitamin C association with PTB** | | | | | | | | | | | |
| **1** | Cohort study | Undetected | No serious inconsistency | No serious indirectness | No serious imprecision | 3,143 | | - | OR 1.55 (1.07-2.24) (vit C) | Low ++/++++ | Important but not critical |
| **Zhang (2017)** [97]**. Plasmatic ascorbic acid levels in PTB** | | | | | | | | | | | |
| **1** | Case control study | Undetected | No serious inconsistency | No serious indirectness | No serious imprecision | 130 | 381 | - | 117.3±60.35 mg (PTB) 127.13±68.17 mg (controls) | Low ++/++++ | Important but not critical |
| **Ilhan (2015)** [15]**, Hassanzadeh (2016)** [93]**. Plasmatic vitamin E levels in PPROM** | | | | | | | | | | | |

| | | | | | | | | | | | |
|---|---|---|---|---|---|---|---|---|---|---|---|
| 1 | Cross-sectional study | Undetected | No serious inconsistency | No serious indirectness | No serious imprecision | 38 | 34 | - | 20.38±6.47 mg/L (PPROM) 18.15±6.38 mg/L (controls) | Low ++/++++ | Of limited importance |
| 1 | Cohort study | Undetected | No serious inconsistency | No serious indirectness | No serious imprecision | 620 | | - | 1.55-2.37 mg/L (PPROM) 1.42-1.71 mg/L (controls) | Low ++/++++ | Of limited importance |
| **Bartfai (2012)** [90], **Harville (2020)** [89], **Carmichael (2013)** [91]. **Vitamin E effects on PTB** | | | | | | | | | | | |
| 1 | Cohort study | Undetected | No serious inconsistency | No serious indirectness | No serious imprecision | 1215 | | - | OR 1.0 | Low ++/++++ | Of limited importance |
| 1 | Case control study | Undetected | No serious inconsistency | No serious indirectness | No serious imprecision | 2,287 | 35,864 | - | OR 0.71 (0.63-0.84) | Moderate +++/++++ | Of limited importance |
| 1 | Transversal study | Undetected | No serious inconsistency | No serious indirectness | No serious imprecision | 5,738 | | - | OR PTB<32 GWs 1.9 (1.0-3.6) | Low ++/++++ | Important but not critical |
| **Zhang (2017)** [97]. **Vitamin E levels in PTB** | | | | | | | | | | | |

| | | | | | | | | | | | |
|---|---|---|---|---|---|---|---|---|---|---|---|
| **1** | Case control study | Undetected | No serious inconsistency | No serious indirectness | No serious imprecision | 130 | 381 | - | 29.6±9.51 mg (PTB) 33.57±11.30 mg (controls) | Low ++/++++ | Important but not critical |
| **Koenig (2017)** [96]. **Vitamin E intake and premature cervical remodeling** | | | | | | | | | | | |
| **1** | Cohort study | Undetected | No serious inconsistency | No serious indirectness | Serious imprecision (small sample size) | 47 | | - | 22.0±22.56 IU (high risk) 45.5±42.86 IU (low risk) | Very low +/++++ | Important but not critical |
| **Gungorduk (2014)** [92]. **Effect of vitamin C+E supplementation to increase latency period before birth in PPROM** | | | | | | | | | | | |
| **1** | RCCT | Serious risk of bias (blinding) | No serious inconsistency | No serious indirectness | No serious imprecision | 126 | 123 | - | 11.2±6.3 days (vit C+E) 6.2±4.0 days (controls) | Moderate +++/++++ | Important but not critical |
| **Hauth (2010)** [94]. **Vitamin C+E and reduction in PPROM+PTB before 32 gestational weeks** | | | | | | | | | | | |
| **1** | RCCT | Undetected | No serious inconsistency | No serious indirectness | No serious imprecision | 4,992 | 4,976 | - | OR 0.5 (0.27-0.93) | High ++++/++++ | Important but not critical |
| **Hauth (2010)** [94]. **Vitamin C+E effect on PTB** | | | | | | | | | | | |
| **1** | RCCT | Undetected | No serious inconsistency | No serious indirectness | No serious imprecision | 4,992 | 4,976 | - | OR 1.05 (0.90-1.22) | High ++++/++++ | Of limited importance |

| | | | | | | | | | | | |
|---|---|---|---|---|---|---|---|---|---|---|---|
| **Zahiri (2015)** [101]**. Zinc and risk of PPROM** | | | | | | | | | | | |
| **1** | RCCT | Undetected | No serious inconsistency | Serious indirectness (different intervention) | No serious imprecision | 270 | 270 | RR 0.08 (Zn) RR 0.07 (controls) | OR 0.09 (Zn) OR 0.07 (controls) | High ++++/++++ | Important but not critical |
| **Kucukaydin (2018)** [103]**, Shen (2015)** [104]**. Plasmatic Zinc levels in PPROM** | | | | | | | | | | | |
| **1** | Case control study | Undetected | No serious inconsistency | No serious indirectness | No serious imprecision | 35 | 38 | - | 0.80±0.30 mg/L (PPROM) 1.76±0.74 mg/L (controls) | Low ++/++++ | Important but not critical |
| **1** | Cohort study | Undetected | No serious inconsistency | No serious indirectness | No serious imprecision | 1,568 | | - | 73.21±13.9 μmol/L (PPROM) 84.55±14.38 μmol/L (controls) | Low ++/++++ | Important but not critical |
| **Nga (2020)** [99]**, Nossier (2015)** [100]**, Zahiri (2015)** [101]**. Zinc use and reduction of PTB** | | | | | | | | | | | |
| **3** | RCCT | Serious risk of bias (blinding) | No serious inconsistency | No serious indirectness | No serious imprecision | 798 | 650 | RR 0.012-0.13 (Zn) RR 0.05-0.10 (controls) | OR 0.01-0.16 (Zn) OR 0.06-0.12 (controls) | Moderate +++/++++ | Important but not critical |
| **Shen (2015)** [104]**. Plasmatic zinc levels in PTB** | | | | | | | | | | | |
| **1** | Cohort study | Undetected | No serious inconsistency | No serious indirectness | No serious imprecision | 1,568 | | - | 73.55±14.09 μmol/L (PTB) 84.55±14.38 μmol/L (controls) | Low ++/++++ | Important but not critical |

| | | | | | | | | | | | |
|---|---|---|---|---|---|---|---|---|---|---|---|
| **Chen (2018)** [105]**, Huang (2016)** [106]**, Lu (2017)** [107]**, Okubo (2015)** [108]**, Moussally (2010)** [109]**, Sengpiel (2013)** [110]**, Sindiani (2020)** [111]**. Tea intake association with PTB** | | | | | | | | | | | |
| **1** | Case control study | Serious risk of bias (unmatched case control) | No serious inconsistency | No serious indirectness | No serious imprecision | 314 | 796 | - | OR 1.43 (>3cups/day) | Very low +/++++ | Important but not critical |
| **6** | Cohort study | Undetected | No serious inconsistency | No serious indirectness | No serious imprecision | 88,381 | | - | OR 0.88-1.61 | Low ++/++++ | Important but not critical |
| **Biran (2019)** [112]**, Ramiro-Cortijo (2020)** [115] **,Specht (2019)** [116]**. Mother plasma melatonin reduced levels at birth.** | | | | | | | | | | | |
| **1** | Cohort study | Undetected | No serious inconsistency | No serious indirectness | No serious imprecision | 169 | | RR 0.78 (<34GWs) RR 0.57 (>34GWs) | OR 3.5 (<34GWs) OR 1.34 (>34 GWs) | Low ++/++++ | Critical |
| **1** | Cohort study | Undetected | No serious inconsistency | Serious indirectness (because of population) | No serious imprecision | 104 | | - | Lower mother melatonin plasmatic levels in PTB (p<0.024). | Very low +/++++ | Critical |
| **Specht (2019)** [116]**. Night work during pregnancy and association with PTB.** | | | | | | | | | | | |
| **1** | Cohort study | Undetected | No serious inconsistency | No serious indirectness | No serious imprecision | 16,501 | | RR 0.05 (night-working) RR 0.05 (day-working) | OR 0.05 (night-working) OR 0.05 (day-working) | Low ++/++++ | Of limited importance |

| | | | | | | | | | | | |
|---|---|---|---|---|---|---|---|---|---|---|---|
| **Zarban (2015)** [121]**, Melo (2017)** [125]**, Medeiros (2016)** [126]**, Clemente (2015)** [135]**. Vitamin C/E and breastfeeding (follow-up of maximum 30 days )** | | | | | | | | | | | |
| **4** | RCCT | No serious risk of bias | No serious inconsistency | No serious indirectness | No serious imprecision | - | - | Supplements of vitamin C and E increase anti-oxidant content of breastmilk, especially in colostrum ($p < 0.05$) | - | High ++++/++++ | Important |
| **Hoppu (2005)** [120]**.Vitamin C and breastfeeding (follow-up of 12 months)** | | | | | | | | | | | |
| **1** | Cross-sectional | Serious risk of bias (no control group) | No serious inconsistency | No serious indirectness | No serious imprecision | 65 | - | Decrease the risk of atopy in high-risk infants ($p < 0.05$) | - | Low ++/++++ | Important |
| **Friel (2007)** [123]**.Vitamin C/iron and breastfeeding (No follow-up)** | | | | | | | | | | | |
| **1** | Experimental | Serious risk of bias | No serious inconsistency | No serious indirectness | No serious imprecision | 65 | - | Iron may provoke oxidative stress in preterm infants and should be divided from vitamin C ($p < 0.05$) | - | Very low +/++++ | Important |
| **Daneel-Otterbech (2005)** [122]**.Vitamin C and breastfeeding (No follow-up)** | | | | | | | | | | | |
| **1** | RCCT | Serious risk of bias | No serious inconsistency | Serious indirectness (different populations) | No serious imprecision | 171 | 142 | Significant increase of AA in human milk after AA supplementation | - | Moderate +++/++++ | Important |

| | | | | | | | | | | | |
|---|---|---|---|---|---|---|---|---|---|---|---|
| | | | | | | | | (1000 mg/d for 10 d) African women: 41 mg/kg ($p < 0.001$) European women: 10 mg/kg ($p = 0.03$) | | | |
| **Loui (2004)** [131], **Shaaban (2005)** [130] **Selenium and zinc and breastfeeding (variable follow-up)** | | | | | | | | | | | |
| **2** | RCCT | No serious risk of bias | No serious inconsistency | No serious indirectness | No serious imprecision | - | - | Zn supplements in lactating women increased breast milk Zn levels ($p < 0.05$)European women: 10 mg/kg ($p = 0.03$) | - | High ++++/++++ | Important |
| **Strambi (2004)** [129]**. Selenium and zinc and breastfeeding (follow-up of 4 weeks)** | | | | | | | | | | | |
| **1** | Longitudinal | No serious risk of bias | No serious inconsistency | No serious indirectness | No serious imprecision | 81 | 129 | Breast-fed SGA higher plasma Se concentrations than formula-fed newborns ($p < 0.05$) | - | Moderate +++/++++ | Important |
| **Qin (2019)** [133]**. Melatonin (follow-up of 30 days)** | | | | | | | | | | | |

| | | | | | | | | | | | |
|---|---|---|---|---|---|---|---|---|---|---|---|
| **1** | Longitudinal | No serious risk of bias | No serious inconsistency | No serious indirectness | No serious imprecision | 32 | 66 | Circadian rhythm in breast milk, highest peak level in colostrum, ($p < 0.05$) | - | Moderate +++/++++ | Important |
| **Honorio-Franca (2013)** [134]. **Melatonin (follow-up of 30 days)** | | | | | | | | | | | |
| **1** | RCCT | Serious risk of bias (no information about randomization) | No Serious inconsistency | Serious indirectness ( no population data at baseline) | Serious imprecision (very wide range of results) | 23 | 24 | No significant effects on IL-10 or COX-2 serum levels | - | Low ++/++++ | Less important |

**Abbreviations:** ARE: antioxidant response element; BP: blood pressure; COX-2: cyclooxygenase-2; EGCG: epigallocatechin gallate; GRADE: Grading of Recommendations Assessment, Development, and Evaluation; GSH: glutathione; GWs: gestational weeks; HC: hypertensive crisis; HO-1: heme oxygenase-1; HUVECs: human umbilical vein endothelial cells; IL-10: interleukin 10; MDA:; NO: nitric oxide; PPROM: preterm premature rupture of membranes; PTB: preterm birth; RCCT: randomized controlled clinical trial; SGA: small for gestational age; Vit: vitamin; Zn: zinc. Quality of evidence grades: high (++++), moderate (+++), low (++), very low (+).
